# Phylogeographic diversity and mosaicism of the *Helicobacter pylori tfs* integrative and conjugative elements

**DOI:** 10.1186/s13100-018-0109-4

**Published:** 2018-01-26

**Authors:** Robin M. Delahay, Nicola J. Croxall, Amberley D. Stephens

**Affiliations:** 1 Nottingham Digestive Diseases Centre and National Institute for Health Research (NIHR) Nottingham Biomedical Research Centre, Nottingham University Hospitals NHS Trust and University of Nottingham, Nottingham, UK; 2 Present Address: Chemical Engineering and Biotechnology, University of Cambridge, Philippa Fawcette Drive, West Cambridge, Cambridge, CB3 0AS UK

**Keywords:** Integrative and conjugative element (ICE), *Helicobacter pylori*, Horizontal gene transfer, *tfs3/tfs4*, Virulence factor, *dupA*, *cag* pathogenicity island, Type IV secretion system, Comparative genomics, Population genomics

## Abstract

**Background:**

The genome of the gastric pathogen *Helicobacter pylori* is characterised by considerable variation of both gene sequence and content, much of which is contained within three large genomic islands comprising the *cag* pathogenicity island (*cag*PAI) and two mobile integrative and conjugative elements (ICEs) termed *tfs3* and *tfs4*. All three islands are implicated as virulence factors, although whereas the *cag*PAI is well characterised, understanding of how the *tfs* elements influence *H. pylori* interactions with different human hosts is significantly confounded by limited definition of their distribution, diversity and structural representation in the global *H. pylori* population.

**Results:**

To gain a global perspective of *tfs* ICE population dynamics we established a bioinformatics workflow to extract and precisely define the full *tfs* pan-gene content contained within a global collection of 221 draft and complete *H. pylori* genome sequences. Complete (ca. 35-55kbp) and remnant *tfs* ICE clusters were reconstructed from a dataset comprising > 12,000 genes, from which orthologous gene complements and distinct alleles descriptive of different *tfs* ICE types were defined and classified in comparative analyses. The genetic variation within defined ICE modular segments was subsequently used to provide a complete description of *tfs* ICE diversity and a comprehensive assessment of their phylogeographic context. Our further examination of the apparent ICE modular types identified an ancient and complex history of ICE residence, mobility and interaction within particular *H. pylori* phylogeographic lineages and further, provided evidence of both contemporary inter-lineage and inter-species ICE transfer and displacement.

**Conclusions:**

Our collective results establish a clear view of *tfs* ICE diversity and phylogeographic representation in the global *H. pylori* population, and provide a robust contextual framework for elucidating the functional role of the *tfs* ICEs particularly as it relates to the risk of gastric disease associated with different *tfs* ICE genotypes.

**Electronic supplementary material:**

The online version of this article (10.1186/s13100-018-0109-4) contains supplementary material, which is available to authorized users.

## Background

Both *Helicobacter pylori* and other non-*pylori Helicobacters* have the capacity to colonise the gastric mucosa of humans, increasing the risk of development of a range of gastrointestinal diseases [1, 2]. In respect of *H. pylori*, these include chronic gastritis, peptic ulcer and gastric adenocarcinoma which account for significant morbidity and mortality worldwide [3]. However, of *H. pylori* infected individuals, estimated to comprise one half of the world’s population, clinical disease manifests in a subset of only 15-20%, less than 1% of which develop gastric cancer [4–6]. This incidence is considered a consequence of the multifactorial nature of infection, in which disease risk and susceptibility is influenced by complex interplay between a variety of host, bacterial and environmental factors [7–9].

*H. pylori* is acquired in infancy, predominantly from within familial or close community groups [10] and invariably maintains a persistent infection throughout the lifetime of the host [4]. The consequent long association with genetically related ethnic groups as a result of such localised transmission has led to the selection of locally adapted *H. pylori* genotypes with distinct phylogeographic signals [11, 12]. These geographic patterns of diversity are concordant with human diversity and have been used in population genetic analyses to define different *H. pylori* populations that co-evolved with human hosts ancestrally native to particular global geographical regions [12–16].

Currently, seven major genetically and geographically distinct *H. pylori* populations (‘hp’) and five subpopulations (‘hsp’) have been described by multi locus sequence typing (MLST) and the STRUCTURE Bayesian population cluster method, defined as hpAfrica1 (subpopulations hspWAfrica, hspSAfrica), hpAfrica2, hpNEAfrica, hpEurope, hpAsia2, hpEAsia (subpopulations hspAmerind, hspEAsia and hspMaori) and hpSahul [13–16]. More recent methods such as fineSTRUCTURE have further resolved the genetic structure of these populations, expanding the number of subdivisions and providing detailed insight of inter-population admixture that contributes to the extreme genetic diversity of *H. pylori* strains [17]. Through such analyses, the association of *H. pylori* with humans is understood to be ancient, significantly pre-dating the migratory expansion of anatomically modern humans out of Africa ca. 60, 000 years ago (60kya) [13, 14, 16, 18, 19]. The long co-evolutionary association with the geographically distinct human populations that subsequently emerged during colonisation of the globe provides a possible explanation for the benign, if not beneficial, lifetime carriage of *H. pylori* by the majority of infected individuals [8, 9, 20, 21] since co-evolution and host adaptation of pathogens, particularly in the context of vertical transmission (parent to child), is proposed to select for reduced virulence and the promotion of commensalism [22]. Consistently, *H. pylori* indigenous to globally remote human populations have been demonstrated to encode an attenuated, low virulence form of the host-interactive CagA oncoprotein [23, 24]. In contrast, events that disrupt the co-evolved complementarity between host and pathogen genotypes, such as horizontal transmission to non-ancestral host populations, may promote more adverse interactions which have consequences for clinical disease [22]. In the absence of discernible correlations with established virulence genotypes, these theories may account for the differing susceptibilities to gastric cancer in populations with similarly high rates of *H. pylori* infection [25–28], and presents discordant host-pathogen co-ancestry as an important co-factor in the determination of disease risk [22].

In addition to the possible competitive displacement of native strains [29, 30], horizontal transmission also provides opportunity for inter-strain DNA transfer and exchange in mixed infection and is a principal mechanism for acquisition of novel genes and virulent genotypes of existing determinants [31, 32]. Both DNA transformation and conjugative processes are considered to contribute to the variability of gene content encoded by different *H. pylori* strains [33–35] with further genomic diversity mediated by high rates of recombination, mutation and phase variation [11, 36–38]. The variable and strain-specific subset of *H. pylori* genes comprises 20-25% of the genome [39, 40] many of which are encoded within three large (>35kbp) horizontally acquired low G + C content chromosomal regions. These are identified as the *cag* pathogenicity island (*cag*PAI) and two distinct ‘plasticity zone’ clusters of genes termed *tfs3* and *tfs4* which both have features common to mobile integrative and conjugative elements (ICEs). These include a subset of genes encoding DNA processing (XerT recombinase involved in ICE excision/integration) and transfer functions (VirD2 relaxase) and the characteristic sequence motifs that direct their activities [41–45], and a complement of type IV secretion system (T4SS) genes with homology to the prototypical *virB/D* T4SS genes of *Agrobacterium tumefaciens* [41], in addition to a variable subset of cargo/accessory genes of unknown function [46, 47]. Although lacking equivalent recombination genes for self-mobility, the *cag*PAI similarly encodes a T4SS known to function in host-cell interaction and translocation of the CagA effector protein. These activities are strongly associated with increased risk of all *H. pylori*-mediated gastroduodenal disease as a consequence of well-established roles in dysregulation of gastric epithelial cell signalling and promotion of sustained host inflammatory responses [5]. A fourth, minimal T4SS encoded by the *comB* locus is present in all *H. pylori* strains and is required for natural DNA transformation competence [33].

Components of the *tfs4* ICE have also been found to associate with varying risk of particular gastroduodenal disease. Most notably, the presence of the gene encoding the putative T4SS VirB4 ATPase (also referred to as duodenal ulcer promoting gene A, or *dupA*), is reported to correlate with an increased risk for development of duodenal ulcer and conversely, reduced risk of atrophy/gastric cancer in some populations [48, 49]. However, that *dupA* disease associations are strengthened when considered in the context of the full *tfs4* ICE is indicative of an important role for other *tfs4*-encoded products in determination of disease risk [50]. Indeed, several other *tfs4* genes (homologues of *H. pylori* strain J99 genes *jhp0947*, *949*, *950*, *951* in particular) are similarly more frequently associated with strains isolated from either or both peptic ulcer disease or gastric cancer disease groups [40, 51–55]. These studies provide compelling evidence of a role for the *tfs4* ICE in *H. pylori*-mediated gastric disease although the identity and functional activity of encoded products relevant to a virulence phenotype remain unknown.

The *tfs3* ICE in contrast has been determined to variably encode the host-interacting pro-inflammatory cell translocating kinase (CtkA) protein [56]. CtkA-mediated stimulation of both host immune and epithelial cell pro-inflammatory signalling suggests it might potentiate gastric mucosal inflammation with consequences for inflammation-associated disease outcomes such as atrophy and gastric cancer [57–59]. Consistently, *ctkA* (encoded by *jhp0940* in strain J99) has been identified as a marker for gastric cancer in some populations [52, 55]. More recently, the *tfs3* ICE-encoded T4SS (Tfs3) has been implicated in promotion of proinflammatory signalling by CtkA [56], although the variable and often low prevalence of *ctkA* in the *H. pylori* (*tfs3*+) strain population [39, 52, 54–57] suggests that the full role of the Tfs3 T4SS in *H. pylori* host interaction remains to be determined.

Rearranged remnant fragments of the *tfs* ICEs were initially identified in a comparison of the first two genome sequenced *H. pylori* strains, 26695 and J99, as components of the strain-specific and variable gene subset [60]. Subsequent studies defined full gene complements and context for representative *tfs3* [41, 42] and *tfs4* ICEs [43] and *tfs4* variation apparent within subsets of complete genome sequences [44, 61, 62]. However, public sequence repositories contain considerably more numerous unfinished, draft whole genome shotgun sequences (WGSs) which, due to their fragmentary nature, have remained refractory to the study of large contiguous chromosomal segments such as ICEs. As these draft assemblies represent an increasingly greater global diversity of *H. pylori* strains, we sought to develop a strategy for efficient data-mining of their constituent, often short read sequence contigs for *tfs* ICE content. Our subsequent analysis of the resulting data-rich resource provides a comprehensive account of *tfs* ICE structure, representation, prevalence and phylogeographic diversity within an extensive global collection of *H. pylori* strains. We show that *tfs* ICEs are modular in their organisation and identify disease-associated accessory modules that are preferentially maintained in strains independently of *tfs* ICE T4SS activity. We further show that population-specific allelic diversity of *tfs3* ICE modules in particular is discriminatory for ICE admixture and inter-population ICE exchange and displacement, and additionally provide evidence for acquisition of an *H. pylori tfs3* ICE by the emerging zoonoses, *H. suis*. These collective events, in addition to the particular modular architecture of different *tfs* ICE types have potential to impact upon different outcomes of persistent infection.

## Results

### Representation of *tfs* ICEs in a global *H. pylori* strain population

Previous descriptions of the *tfs* ICEs of *H. pylori* have predominantly resulted from examination of contiguous ICE clusters in a limited number of available complete genome sequences [44, 61, 62]. However, data-mining of substantially more numerous draft genome sequences in this context has been largely overlooked, not least because of difficulties in establishing full representation and context of large ca. 35-55 kb genomic segments which are invariably distributed over a variable number of non-sequential, often short read contigs. That *tfs* ICEs may also occur in a fragmented state or concurrent with additional *tfs* ICEs or additional partial segments further confounds contextual analysis. To address this, we developed a robust bioinformatics workflow to enable identification and contextual reconstruction of *tfs* ICE clusters encoded within draft WGS sequences. Initially, selected reference *tfs* ICE clusters were manually re-annotated to determine the full complement of *tfs* genes and resolve disparities in the often variable definition of coding sequence arising from automated annotation. A subset of 59 distinct *tfs* gene sequences resulting from this analysis were translated and subsequently used in a sequential BLASTp interrogation of the PATRIC RefSeqProt database, then search hits, comprising > 12,000 *tfs* genes extracted from 221 complete and draft genomes, manually compiled into an ordered dataset (Additional file 1). Sequences were tagged as being *tfs3*, *tfs4* or *com*-encoded by identification of conserved sequence motifs, then full *tfs* representation determined sequentially for each strain genome. Complete and remnant *tfs* clusters were finally assembled by reference to the original re-annotated reference ICE clusters as necessary (Additional file 2). The resulting dataset provides a comprehensive assessment of the global prevalence, structure and allelic frequency of the *tfs* elements both in the context of each other and *cag* status in an extensive collection of *H. pylori* strains, representing 7 phylogeographic populations isolated from 23 different countries.

Comparison of reconstructed *tfs* ICE clusters identifies a core subset of 21 putative genes broadly conserved between both *tfs3* and *tfs4* ICEs (denoted t3/t4_C1-C21) and a further 19 genes unique to each (denoted t4_V1-V19 and t3_V20-V38 for *tfs4* and *tfs3* respectively) (Table 1). Within both core and variable gene subsets, two distinct variants of multiple *tfs4* genes can be identified distributed along the entire length of the *tfs4* ICE, broadly contained within each of two left, central and right segments, referred to here for brevity as L1/L2, C1/C2 and R1/R2 respectively (Table 1, Additional file 2). The *tfs3* ICE in contrast, comprises a more modest subset of variable genes clustered within the left segment of the ICE. Accounting for these, the *tfs4* and *tfs3* pangene complements are determined to comprise 54 and 42 distinct genes/orthologues respectively.

**Table 1:** Modular complements of *tfs* ICE conserved and variable genes in reference *H. pylori* genomes

				*tfs* ICE present in reference *H. pylori* genomes and gene annotation^a^				
				*tfs4*			*tfs3*	
*tfs* gene assignment^b^ (t3_ or t4_)	*tfs* segment	Gene homologue	*pz* homologue^c^	P12 (L2C1R2)	Shi470 (L1C1R1)	SouthAfrica7 (LmC2R2)	Gambia 94/24	India7
**C1.1/C1.2**	*tfs4* Left *(*L1/L2) and *tfs3* Left segment	***xer***	**40**	**437**	**4480**	**7710**	**7345**	**3725**
V1		*–*	37	438	–	7705	7360	NA
**C2.1/C2.2**		***virB6***	**34**	**439**	**4485**	**7700**	**7375**	**3760**
V2		*–*	–	440	–	7695	–	–
**C3.1/C3.2**		***–***	**35**	**441**	**4490**	**7690**	**7370**	**3755**
**C4.1/C4.2**		***–***	**15**	**442, 443**	**4495**	**7685**	**7480**	**3850**
**C5.1/C5.2**		***–***	**32**	**444**	**4500**	**NA**	**7385**	**3770**
**C6.1/C6.2**		***–***	**31**	**446**	**4505**	**7670**	**7390**	**3775**
V3		*–*	–	NA	–	–	–	–
V4	*tfs4* Central (C1/C2) and *tfs3* Left and Right segments	methylase	21	447	4510	7665	NA	3820
**C7**		***virC1***	**29**	**448**	**4515**	**7660**	**7400**	**3785**
**C8**		***–***	**28**	**449**	**4520**	**7655**	**7405**	**3790**
V5		*–*	–	450	4525	–	–	–
**C9.1/C9.2**		***virD2***	**41**	**451**	**4530**	**7650**	**7340**	**3720**
V6		*–*	–	–	–	7640	–	–
V7		*–*	–	452	4535	–	–	–
V8		*–*	–	453	4540	–	–	–
**C10.1/C10.2**		***virD4***	**20**	**454**	**4545**	**7635**	**7455**	**3825**
V9		*–*	20	455	4550	7635	–	–
V10		*–*	–	456	4555	–	–	–
V11		*–*	–	–	–	7630	–	–
V12.1/V12.2		*–*	–	457	4560	7625	–	–
**C11.1/C11.2**		***virB11***	**18**	**458**	**4565**	**7620**	**7465**	**3835**
V13		*–*	–	459	4570	–	–	–
V14		*–*	–	–	–	7615	–	–
V15		*–*	–	460	4575	–	–	–
V16		*–*	–	–	–	7610	–	–
V17		*–*	–	461	4580	7605	–	–
V18		*–*	–	–	4585	–	–	–
**C12**		***virB10***	**14**	**462**	**4590**	**7600**	**7485**	**3855**
**C13**		***virB9***	**13**	**463**	**4595**	**7595**	**7490**	**3860**
**C14.1/C14.2**	*tfs4* Right *(*R1/R2) and *tfs3* Right segment	***virB8***	**12**	**464**	**4600**	**7590**	**7495**	**3865**
**C15.1/C15.2**		***virB7***	**11**	**465**	**4605**	**NA**	**7500**	**3870**
**C16.1/C16.2**		***topA***	**24**	**466**	**4610**	**7585, 7570**	**7425**	**3810**
**C17.1/C17.2**		***virB4***	**10**	**467**	**4615**	**7565**	**7505**	**3875**
**C18.1/C18.2**		***virB3***	**8**	**468**	**4620**	**7560**	**7515**	**3885**
**C19.1/C19.2**		***virB2***	**7**	**469**	**4625**	**7555**	**7520**	**3890**
**C20.1/C20.2**		***–***	**33**	**471**	**4630-35**	**7550**	**7380**	**3765**
V19		*–*	–	472	–	7545	–	–
**C21.1/C21.2**		***–***	**5**	**473**	**4640**	**7540**	**7535**	**3900**
V20	*tfs3* Left segment	*–*	39	–	–	–	7350	3730
V21		*–*	38	–	–	–	7355	3735
V22.1/V22.2		*fic*	–	–	–	–	–	–
V23		*ctkA*	–	–	–	–	–	–
V24.1-V24.4		*–*	36	–	–	–	7365	3750
V25.1-V25.3		*–*	30	–	–	–	7395	3780
V26		–	27	–	–	–	7410	3795
V27		–	26	–	–	–	7415	3800
V28		–	25	–	–	–	7420	3805
V29	*tfs3* Central variable	–	23	–	–	–	7430	3815
V30		–	–	–	–	–	NA	–
V31		–	22	–	–	–	7435	–
V32		–	–	–	–	–	7440	–
V33	*tfs3* Right segment	–	19	–	–	–	7460	3830
V34		–	17	–	–	–	7470	3840
V35		–	16	–	–	–	7475	3845
V36.1-V36.3		–	9	–	–	–	7510	3880
V37		–	6	–	–	–	7525	3895
V38		–	–	–	–	–	7530	NA

Bold text highlights conserved *tfs3/4* homologous genes

*NA* ‘not annotated’ in reference genome

^a^Automated gene numbering increases in multiples of either 1 or 5 (strains Shi470, PeCan4 and SouthAfrica7) in the Genbank annotation of the selected genome sequences

^b^Nomenclature used in the study for reference to *tfs* ICE genes. *tfs3* or *tfs4* (t3_ or t4_) genes are assigned a C or V prefix depending on whether they are (C) conserved in all *tfs* ICE types, or are (V) variably present, as defined by absence in at least one *tfs* ICE type. Numbering proceeds as genes are encountered in order, first from left to right of *tfs4*, then left to right of *tfs3*. Distinct alleles of any particular *tfs* gene are indicated by additional numbering after the period, for example t4_C9.1 and t4_C9.2 represent two distinct alleles of the *tfs4 virD2* gene

^c^*pz* nomenclature initially used in the early description of the *tfs3* ICE from strain PeCan18b [41]

**Fig. 1:**
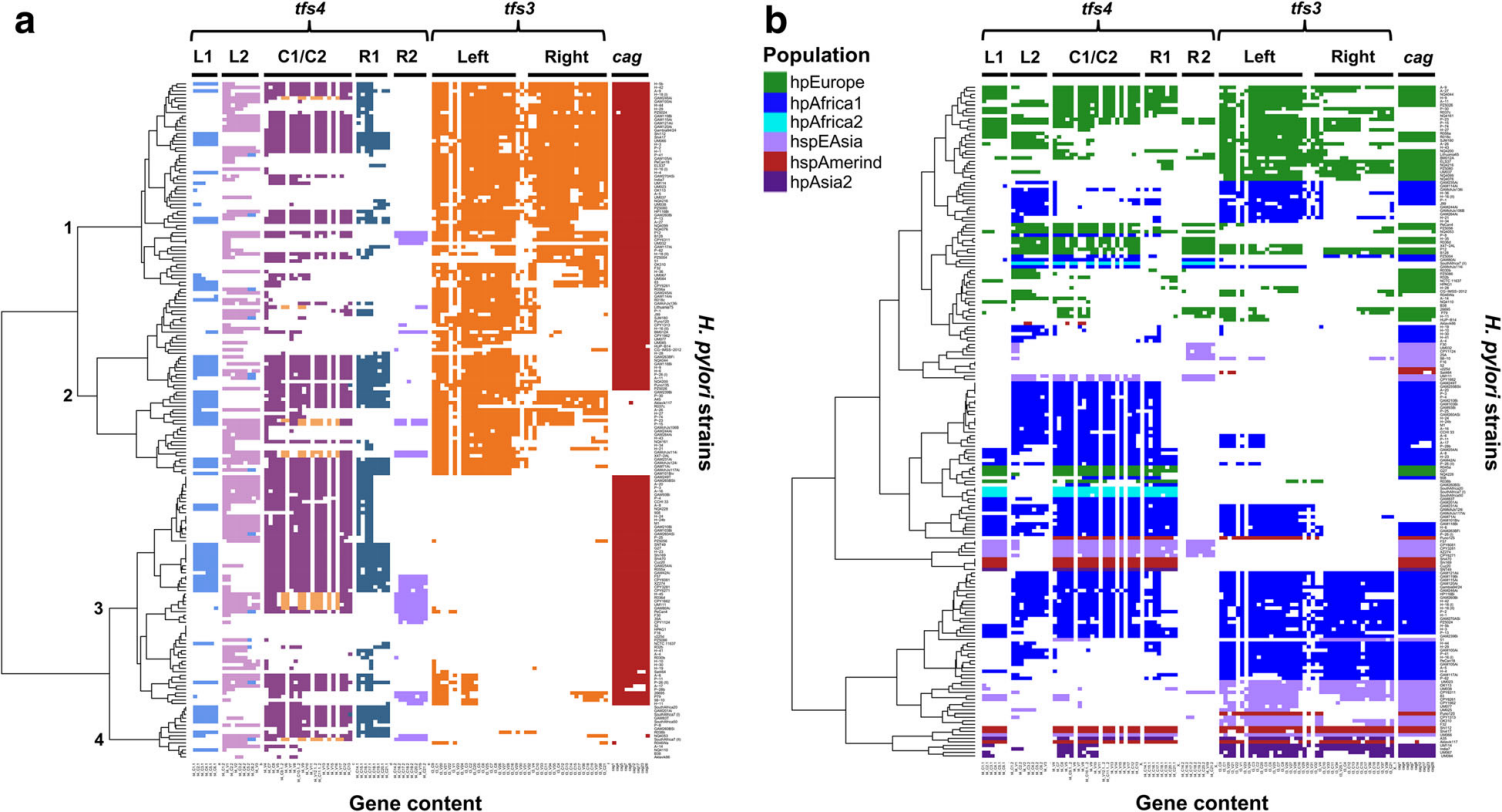
Prevalence of *tfs* ICE and selected *cag* genes in 187 *H. pylori* genomes (for a high resolution version of this figure, please see Additional file 6). **a** Clustering defines the strain representation and integrity of *tfs* ICEs relative to each other and *cag*PAI status. Distinct clusters (1–4) identify strains with similar representation of *tfs* and *cag* gene complements. Colouring of *tfs4* gene subsets corresponds to different complements of orthologous genes contained within each of two left (L1 and L2), central (C1 and C2) or right (R1 and R2) ICE segment modules which may be contiguous with, isolated from or additional to the tri-module complement of a particular *tfs4* ICE type. The *tfs3* ICE lacks an equivalent pattern of modular variation and is more broadly defined by left and right segments. **b** *tfs* ICE and *cag* gene content data was transformed to reflect phylogeographic lineage of the originating strain as determined by MLST relative to a subset of 347 reference sequences obtained from the MLST database (Additional file 3: Figure S1). *tfs* and *cag* gene complements were coloured according to the phylogeographic population assigned to each strain, as indicated in the inset key. Hierarchical clustering identifies extensive erosion of the *tfs* ICEs in hpEurope strain populations

Hierarchical clustering of the *tfs* and *cag* datasets based on gene content contained within particular segments (Fig. 1a) highlights the relative ubiquity of the *tfs4* ICE, either as a complete (96 strains) or fragmented (74 strains) cluster compared to either *tfs3* or *cag* which, although broadly prevalent in the population, are entirely absent in 60 and 37 strains respectively and concurrently so in 11. Notably, *tfs3* and *tfs4* are maintained together in substantially intact form in 18 strains although commonly, the right end segment of the *tfs3* ICE encoding the majority of putative T4SS genes is absent (53 strains with partial clusters compared with 43 containing intact *tfs3* ICEs).

Transformation of the data to enable clustering on the basis of phylogeographic population highlights an apparent increase in fragmentation of both *tfs* ICEs in strains of European (hpEurope), and to a lesser extent, African (hpAfrica1) descent (Fig. 1b) suggesting that the ICEs may be less stable and/or more dispensable in these backgrounds. No correlation between presence, absence or co-occurrence of intact *tfs* and *cag* clusters could be determined suggesting that the clusters are independent of each other. Although *tfs3*/*tfs4* co-carriage appeared less common in the hspEAsia population (Fig. 1b), there was a similar lack of correlation between the presence of intact/remnant *tfs3* or *tfs4* ICEs suggesting that neither ICE presents a strict barrier to acquisition or stable maintenance of the other. In view of this, it might therefore be considered that the encoded functions of the *tfs3*/4 ICEs are also unlikely to be antagonistic.

### Modular disposition of the *tfs4* ICE and hybrid *tfs4* ICE types

Up to three *tfs4* ICE types have been previously described from comparative analysis of complete genome sequences based on the presence of particular orthologous gene subsets, [42, 62], most recently referred to as ICEHp*tfs4*a-c [44]. Hierarchical clustering of *tfs4* ICE gene content similarly identifies these types, in which distinct orthologous gene complements are precisely contained within L2-C1-R2 (ICEHp*tfs4*a), L1-C1-R1 (ICEHp*tfs4*a) and L1/L2-C2-R2 (ICEHp*tfs4*c) ICE segments (Table 1, Additional file 3: Figure S2, clusters 1 and 3). This assessment reveals that *tfs4* ICEs have a distinct modular organisation in which each of two variant L-C-R modules may be interchangeable in the generation of a particular *tfs4* type. Interestingly, L1 and L2 left flank modules are occasionally observed in addition to and in isolation from both complete and remnant ICE clusters (Additional file 3: Figure S2, cluster 4). This is similarly observed, albeit to a lesser extent, with R1 and R2 right flank modules, although only the R2 module is apparent in isolation from other sections of the *tfs4* ICE (Additional file 3: Figure S2, cluster 3). Similar to the R2 module, the C2 central module is present in only a small subset of strains, invariably concurrent with the R2 flank.

**Fig. 2:**
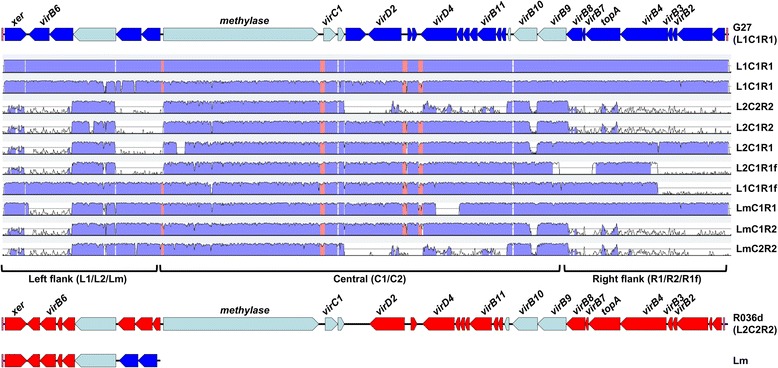
Global pairwise sequence alignment of distinct *tfs4* ICE modular subtypes. The ‘*vir*’ gene labelled *tfs4* ICE gene cluster (top) is representative of the *tfs4* ICE from strain G27 (L1C1R1, including genes 0959–0986) used as a reference scaffold sequence for each ICE alignment and closely approximates the position of genes in the alignment graphs. L1, C1 and R1 module gene complements are coloured blue, whereas orthologous genes characteristic of L2, C2, R2 modules are shown coloured red in the schematic of the L2C2R2 ICE type from strain R036d (bottom). Sequence conserved genes common to all *tfs4* ICEs are coloured cyan. Global pairwise sequence alignments of different *tfs4* ICEs was performed using the Shuffle-LAGAN alignment program in mVISTA. Individual mVISTA graphs depict sequence similarity of *tfs4* ICEs from strains Shi470 (L1C1R1, genes 4480–4640), R036d (L2C2R2, genes 0976–1010), P12 (L2C1R2, genes 0437–0473), NQ4053 (L2C1R1, genes 1511–1596), P-25 (L2C1R1f, genes 1401–1438), A-8 (L1C1R1f, genes 0913–0945), A-11 (LmC1R1, genes 0721–0756), PeCan4 (LmC1R2, genes 7670–7835) and SouthAfrica7 (LmC2R2, genes 7540–7710) relative to the G27 *tfs4* ICE using a 50 bp calculation window and 70% sequence identity cut-off. The midline in individual alignment graphs corresponds to 50% sequence identity to the G27 reference *tfs4* ICE sequence and regions with sequence identity ≥ 70% are indicated by blue (coding sequence) or red (non-coding intergenic sequence) shading

Scrutiny of L1/L2-C1/C2-R1/R2 module representation in the context of contiguous gene content identifies the L1C1R1 modular configuration to be predominant in the *H. pylori* population, with 37 genomes harbouring a complete L1C1R1 ICE (Table 2). A second, previously undescribed configuration, L2C2R2 can be discerned intact in a further 9 genomes. Two rare configurations L2C1R2 (ICEhp*tfs4*a in [44], 2 strains) and L2C1R1 (2 strains) can also be identified in addition to several others which incorporate either a truncated R1 module fragment (‘R1f’), or a mosaic left flank (‘Lm’) module comprising five and two L2 and L1 genes respectively flanking the L1/L2 conserved gene t4_C4 (Table 2), equivalent to ICEHp*tfs4*c [44]. As illustrated by comparative global sequence alignment of each defined *tfs4* ICE type (Fig. 2), both Lm and R1f flanks can associate with any modular organisation suggesting that they both represent stable modular types rather than constructions arising repeatedly through occasional recombination or aberrant ICE integration. A pairwise sequence comparison between the modular complements of encoded orthologous *tfs* T4SS proteins, and that of *cag* and *com*, further emphasises the distinctiveness of the different elements (Additional file 4: Table S1a and [44]).

**Table 2:** Modular composition and properties of defined *tfs4* ICE types in reference strains

						Annotated genes contained within particular variant L-C-R modules^a^		
Strain^b^	ICE*Hp* type^c^	ICE type (L-C-R)^d^	No. intact^e^	ICE size^f^ (bp)	Position in genome	L1/L2/Lm	C1/C2	R1/R2/R1f
G27	*tfs4b*	L1C1R1	37	39,129	1,045,701-1,085,076	986-981	980-966	965-959
Shi470	*tfs4b*	L1C1R1		39,376	874,706-913,876	4480-4505	4510-4595	4600-4640
R036d	*–*	L2C2R2	9	39,125	403,599-442723^g^	1010-1000	999-985	984-976
P12	*tfs4a*	L2C1R2	2	40,644	452,130-492,773	437-446	447-463	464-473
NQ4053	*–*	L2C1R1	2	40,414	338,351-378764^h^	1511-1502	1501-1476	1590-1596
P-25	*–*	L2C1R1f	34	35,201	685,842-721042^i^	1438-1425	1424-1406	1405-1401
A-8	*–*	L1C1R1f	3	35,548	275,596-311143^j^	945-939	938-919	917-913
A-11	*–*	LmC1R1	2	38,543	689,499-728042^k^	756-748	747-729	728-721
PeCan4	*tfs4a*	LmC1R2	1	40,987	1,537,078-1,578,064	7835-7800	7785-7710	7705-7670
SAfrica7	*tfs4c*	LmC2R2	1	41,220	1,527,377-1,568,596	7710-7670	7665-7595	7590-7540

^a^Automated gene numbering increases in multiples of either 1 or 5 (strains Shi470, PeCan4 and SouthAfrica7) in the Genbank annotation of the selected genome sequences

^b^Representative reference strains harbouring different defined *tfs4* ICE types (with the exception of G27/Shi470) for which contiguous ICE sequences are available

^c^As defined in [44]

^d^L-C-R refers to particular types of left (L1/L2/Lm), central (C1/C2) or right (R1/R2/R1f) *tfs4* ICE modules comprising different subsets of orthologous genes

^e^Intactness of different *tfs4* ICE types in strains based on the relative representation of component modules

^f^Total length of *tfs4* ICE sequences between left and right flanking *xer* excision motifs as defined in [42]

^g^HpR036dcontig.3, accession: NZ_AMOT01000004.1

^h^NQ4053contig.5_1, accession NZ_AKNV01000006.1

^i^HpP_25.contig.1, accession AKPS01000002.1

^j^HpA_8.contig.3_1, accession AKOS01000004.1

^k^HpA_11.contig.0, accession NZ_AOTW01000001.1

Since L1C1R1 and L2C2R2 modular organisations are predominant in the *H. pylori* population and account for all orthologous and unique *tfs4* genes, they likely represent two principal ancestral *tfs4* ICE types. Other *tfs4* module configurations are therefore presumably hybrid derivatives which conceivably arose as a consequence of partial integration/exchange during heterologous ICE transfer or genome rearrangement and recombination between two resident ICEs.

### Phylogeographic distribution of *tfs4* ICEs and component modules

On the basis of MLST, *H. pylori* strains can be assigned to different phylogeographic populations which are informative of their geographical and ancestral origins. To determine if phylogeographic origins of different *tfs4* ICE types could be discerned, hierarchical clustering was performed on the *tfs4* gene dataset in the context of host strain lineage. The analysis clearly demonstrated the presence of the L1C1R1 ICE in all phylogeographic groups (Additional file 3: Figure S3), of which, a significantly increased prevalence was apparent for hpAfrica2, hpAfrica1 and hspAmerind populations (Additional file 4: Table S2). By contrast, the L2C1R1f type appeared to be exclusive to either hpAfrica1 (*p* < 0.0001) and to a lesser extent, hpEurope strain lineages. Notably, all hybrid *tfs4* ICEs with the single exception of LmC2R2, were only found in hpAfrica1/hpEurope strain populations in which the L2C2R2 ICE was also most prevalent (Additional file 4: Table S2). Hybrid ICEs are therefore indicated to have arisen from module exchange between the two predominant *tfs4* ICEs following co-infection with these particular strain populations.

**Fig. 3:**
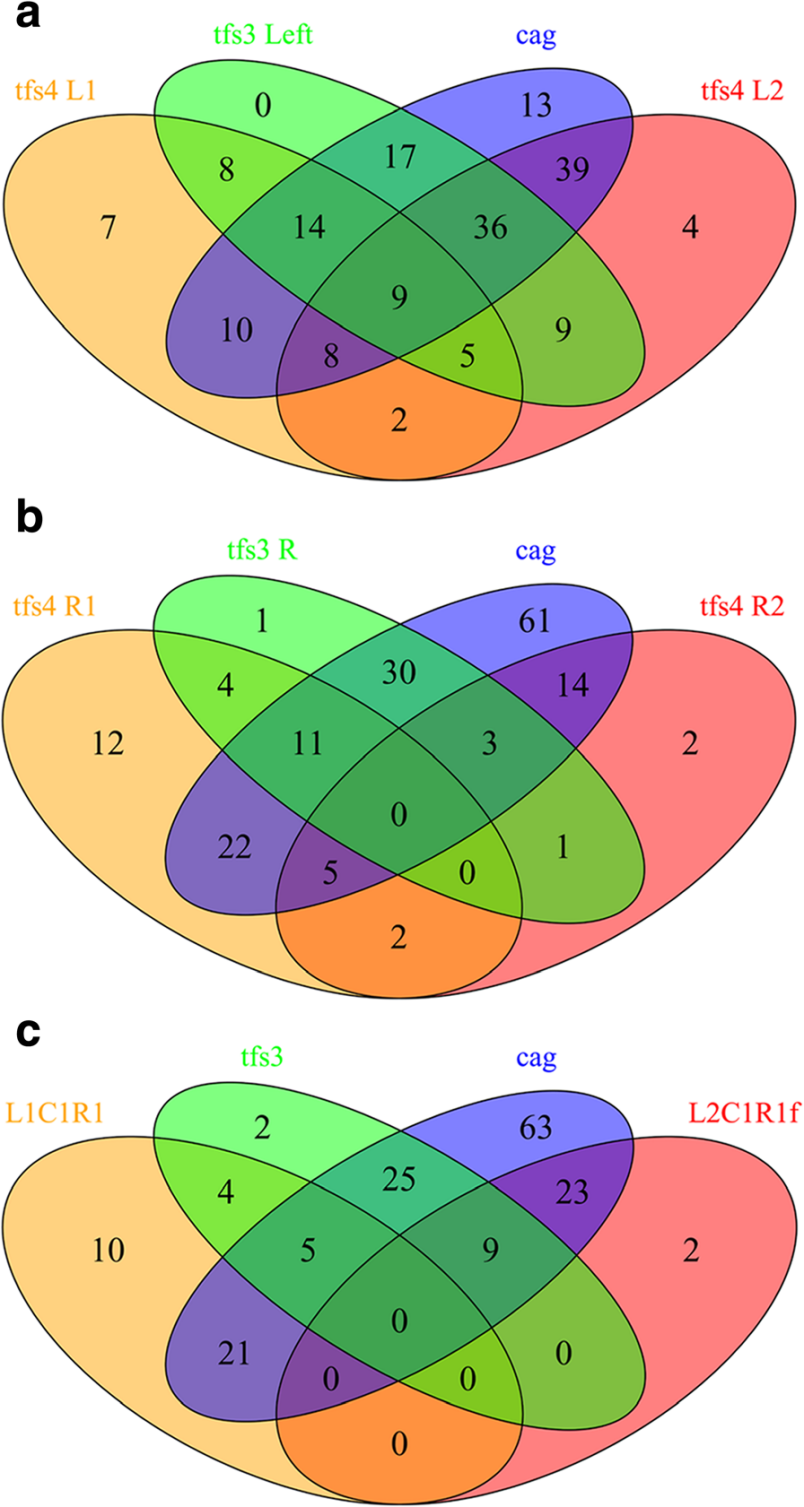
Venn diagrams showing co-occurrence of *tfs* ICE modules/types with each other and the *cag*PAI. **a** Non-T4SS accessory/cargo *tfs* genes located within *tfs4* L1/L2 modules and the *tfs3* left segment. **b** *tfs4* R1/R2 modules and the *tfs3* right segment comprising the majority complement of *tfs* T4SS transfer genes. **c** Intact *tfs3* and L1C1R1 and L2C1R1f *tfs4* ICE types. Plots a. and b. highlight a complex history of *tfs* ICE/module acquisition in which cargo/accessory modules (a) are more commonly maintained in strain genomes compared with transfer modules (b). Erosion of transfer modules is further reflected by the low co-occurrence of intact *tfs3* with either of the most prevalent *tfs4* types, L1C1R1 (*dupA*+) or L2C1R1f (partial *dupA*), co-resident with *tfs3* in 22 and 26% of strains respectively (c). Although most commonly co-resident with the *cag*PAI, all *tfs* modules and ICE types were also apparent in strains which lacked it. Representation of intact elements was determined in 181, 168 and 164 *H. pylori* genomes (a-c respectively). *tfs4* ‘Lm’ hybrid left flanks were included with L2 counts in a. and L2C1R1f counts in c

Given the apparent propensity of *tfs* ICEs for modular exchange and the relative abundance of individual L-C-R modules in the global strain population compared to intact ICEs, we also considered the distribution of modules independently of a particular ICE architecture. This analysis was more informative of previous ICE carriage and the population skew of L/R modules in particular. Consistent with findings for the L2C1R1f ICE, L2 and R1f modules were most commonly associated with the hpAfrica1 lineage (*p* < 0.0001). Similarly, findings for the R1 module reciprocated the association of the L1C1R1 ICE with hpAfrica2/hspAmerind lineages, whereas the R2 module was significantly more prevalent in hspEAsia strains (Table 3). As with L1C1R1 type ICEs in general, the L1 module was found to be widely distributed, but particularly prevalent in hpAfrica1/2 and hpAsia2 populations. This widespread but variable distribution of *tfs4* ICEs and component modules is further emphasised by an assessment of module co-occurrence in strains, revealing a complex history of *tfs* ICE acquisition, exchange and erosion (Fig. 3a and b) which is likely to make a notable contribution to strain diversity.

**Table 3:** Phylogeographic distribution of individual *tfs4* modules

		Prevalence (%) of *tfs4* modules in *H. pylori* populations							
Population	Strains	L1	L2	Lm	C1	C2	R1	R2	R1f
hpEurope	53	20 (38)	21^b^ (40)	7^e^ (13)	32 (60)	6 (11)	13 (25)	12 (23)	8^b^ (15)
hpAfrica1	86	19^b^ (22)	69^d^ (80)	5 (6)	57 (66)	3 (3)	17 (20)	2^d^ (2)	49^d^ (57)
hpAfrica2	3	3^a^ (100)	–	1 (33)	3 (100)	1 (33)	3^a^ (100)	1 (33)	–
hpAsia2	6	5^a^ (83)	1 (17)	–	5 (83)	–	2 (33)	–	–
hspEAsia	28	9 (32)	12 (43)	–	9^a^ (32)	2 (7)	6 (21)	14^d^ (50)	–
hspAmerind	11	6 (55)	–	–	7 (64)	–	7^b^ (64)	–	–
Totals	187	62 (33)	103 (55)	13 (7)	106 (56)	12 (6)	48 (26)	29 (16)	57 (30)

*P* value was determined by Fisher’s Exact Test and indicates significant association of a *tfs4* cluster region with a particular *H. pylori* phylogeographic population

^a^*P* < 0.05

^b^*P* < 0.01

^c^*P* < 0.001

^d^*P* < 0.0001

^e^*P* = 0.051

With further respect to the R1f module, examination of the L2C1R1f genomic context identified the ICE to be invariantly integrated adjacent to 23 s-5 s RNA genes at the L2 left flank end and proximal to *ftsZ-ftsA* and a gene encoding a mechanosensitive ion channel domain-containing protein at the truncated R1f right flank. Characteristically, a gene homologous to *jhp0914* from strain J99 is also uniquely present in this context in hpAfrica1 strains [39, 44]. This strict conservation of integration site, apparent in 57/58 genomes with an R1f module in this study (Additional file 2), restricted distribution and deficit of T4SS assembly genes presumably employed in conjugative ICE transfer, suggests that the L2C1R1f *tfs4* subtype is likely to be immobile and therefore fixed in the hpAfrica1 strain population, conceivably arising from a single ancestral strain in which the R1f truncation originally occurred. Further support for this notion is provided by the observation that the great majority of strains with the L2C1R1f ICE (94%) are also *cag*PAI+ (Fig. 3c) in marked contrast to the prototypical L1C1R1 and L2C2R2 types (68 and 55% co-resident with the *cag*PAI respectively) which can be presumed to mobilise between more genetically diverse host strain populations. Unlike the observed disparity in *cag*PAI co-occurrence however, both L1C1R1 and L2C1R1f *tfs4* types are notably most frequently observed in the absence of a complete, T4SS-competent *tfs3* ICE (22 and 26% co-occurrence with *tfs3* respectively).

Interestingly, a remarkably conserved partial *tfs4* C1 module comprising genes t4_C7/C8/V5 flanked by remnants of t4_V4 (methylase) and t4_C9.1 (*virD2*) is also present in *H. acinonychis* str. Sheeba (Additional file 2). *H. acinonychis* is estimated to have diverged from the *H. pylori* hpAfrica2 super-lineage following a single host jump from humans to large felines 43–56,000 years ago [16, 18]. This suggests that the association of *tfs4* with *H. pylori* is ancient and further, that the C1 module was part of an ancestral *tfs4* ICE, which would reasonably account for its ubiquity in the current global *H. pylori* population.

### Architecture and allelic diversity of the *tfs3* ICE

Although apparent in all *H. pylori* populations, except hpAfrica2 in which the *cag*PAI is also absent, the overall distribution of the *tfs3* ICE in strains is notably reduced in comparison with *tfs4* (Fig. 1, 123 vs 170 strains respectively with evidence of *tfs* acquisition). It is also more commonly present as an incomplete cluster (intact: fragmented ratio of 1:8 and 1:3 for *tfs3* and *tfs4* respectively) although fragmentation characteristically entails loss (right segment) and retention (left segment) of defined ICE segments (Fig. 1, Additional file 3: Figure S4). In the examined dataset, the left segment is present in 78% of all strains with some form of *tfs3* ICE and further enriched in hspEAsia/hpAsia2 and hpAfrica1 *H. pylori* populations (Table 4). Notably, in all strains in which it occurs, *tfs3* and its more highly represented left segment appear co-resident with the more ubiquitous *tfs4* L modules and/or the *cag*PAI (Fig. 3a) suggesting potential differences in stability or temporal acquisition of *tfs3* compared with these other genomic elements.

**Table 4:** Status of *tfs3* ICEs in different *H. pylori* phylogeographic populations

		*tfs3* ICE representation (%) in *H. pylori* populations					
Population	Strains (*tfs3*+)	Intact	Absent	Left flank only^a^	Fragmented (other)	Left flank total^b^	Left flank total (*tfs3*+ only)
hpEurope	53 (42)	8 (15)	11 (21)	17 (32)	17 (32)	25 (47)	25 (59)
hpAfrica1	86 (55)	24 (28)	31 (36)	24 (28)	7 (8)	48 (56)	48 (87)
hpAfrica2	3 (0)	–	3 (100)	–	–	–	–
hpAsia2	6 (5)	3 (50)	1 (17)	2 (33)	–	5 (83)	5 (100)
hspEAsia	28 (15)	5 (18)	13 (46)	8 (29)	2 (7)	13 (46)	13 (87)
hspAmerind	11 (6)	3 (27)	5 (45)	2 (18)	1 (9)	5 (45)	5 (45)
Totals	187 (123)	43 (23)	64 (34)	53 (28)	27 (14)	96 (51)	96 (78)

^a^Left flank defined as spanning genes t3_C9 to t3_V25 inclusive

^b^‘Left flank only’ plus ‘Intact’

**Fig. 4:**
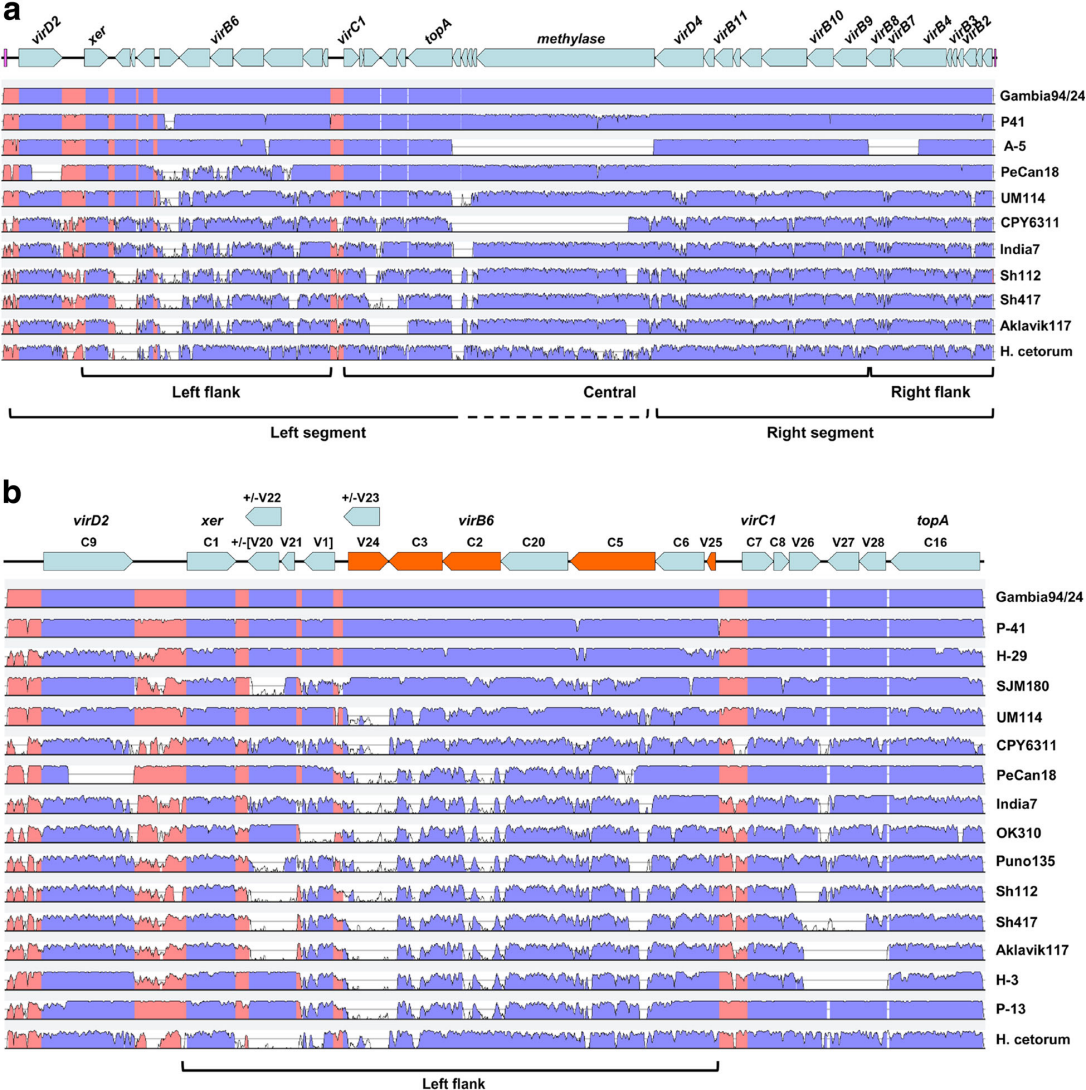
Allelic diversity of the *tfs3* ICE. **a** The ‘*vir*’ gene labelled *tfs3* ICE gene cluster (top) is representative of the complete *tfs3* ICE from strain Gambia94/24 (genes 7340–7535) used as reference for the ICE alignments and closely approximates the position of genes in the alignment schematic. Individual mVISTA graphs depict sequence similarity of *tfs3* ICEs from strains P-41 (genes 0159–0204), A-5 (genes 1146–1181), PeCan18 (genes 4840–5050), UM114 (genes 1250–1445), CPY6311 (genes 0113–0153), India7 (genes 3720–3900), Shi112 (genes 1185–1415), Shi417 (genes 7490–7655), Aklavik117 (genes 2160–2315) and *H. cetorum* strain MIT 00–7128 (genes 8040–8260) relative to the Gambia94/24 *tfs3* ICE in global pairwise sequence alignments using a 50 bp calculation window and 70% sequence identity cut-off. Labelled brackets indicate broad positional conservation of similar gene groupings as the *tfs4* ICE. **b** Extent of sequence and gene content variability within the left flank of the *tfs3* ICE. (Top) Schematic shows the complement of 20 genes in the left flank of the *tfs3* ICE (*virD2* [C9] to *topA* [C16] inclusive) annotated according to the nomenclature described in Table 1. Highly variable genes are block highlighted in orange. Relative position of genes closely approximates corresponding positions in the alignment graphs (bottom). Individual mVISTA graphs depict sequence similarity of *tfs3* ICEs from 16 strains including strains P-41, H-29, SJM180, UM114, CPY6311, PeCan18, India7, OK310, Puno135, Shi112, Shi417, Aklavik117, H-3, P-13 and *H. cetorum* strain MIT 00–7128 relative to the Gambia94/24 *tfs3* ICE (genes 7340–7425) using a 50 bp calculation window and 70% sequence identity cut-off. For both a. and b., the mid-line in individual alignment curves corresponds to 50% sequence identity to the Gambia94/24 reference *tfs3* ICE sequence and regions with sequence identity ≥ 70% are indicated by blue (coding sequence) or red (non-coding intergenic sequence) shading. Gaps indicate absent genes

Although *tfs3* lacks the overt modular disposition of *tfs4* illustrated in Fig. 2, similar left, central and right segments of the ICE which comprise broadly equivalent gene subsets can be defined (Fig. 4a). With the exception of several variably present central genes, t3_V29/V30/V31/V32/V4, central and right flank regions encoding the majority of T4SS assembly genes, are well conserved in both gene content (Fig. 4a) and sequence identity (> 85% nucleotide sequence identity for the majority of genes examined, Additional file 4: Table S3). *tfs3* left flanks by contrast, are hypervariable in these respects, markedly differing in composition (selective gain/loss of genes t3_V1/V20/V21/V22/V23 as previously highlighted [56]) and possession of multiple distinct variants of genes t3_V24/C3/C2/C5 and t3_V25 in particular (Additional file 4: Table S3 and Fig. 4b). Within this variable subset, variation of the t3_C5 genes is distinctive, comprising 5′, 3′ and central regions of conserved sequence interspersed with more highly variable segments differing both in size and sequence composition.

With the exception of t3_C5, Neighbour-Joining phylogenetic trees identify 2–5 distinct clades for each of these genes (illustrated in Additional file 3: Figure S5). However, divergence of the distinct *tfs3* alleles is more modest compared with the substantial separation observed for the corresponding *tfs4* orthologues. Phylogeographic origins can be clearly discerned for the two most prevalent alleles of each gene in the *tfs3* variable subset (annotated as ‘1’ and ‘2’), invariantly corresponding to hpAfrica1/hpEurope or hpAsia2/hspEAsia populations respectively. Remaining distinct clades (‘3-5’), more distantly related to either of the two main clades, comprise more mixed populations but characteristically include sequences from hspAmerind isolates (Table 5). These latter include strains Shi112 and Shi417 isolated from residents of the remote Peruvian Amazonian village of Shimaa, which together with other Shimaa isolates are known to fall within a unique phylogenetic cluster more distantly related to other *H. pylori* populations [30]. The population heterogeneity of these ostensibly hspAmerind clades may therefore reflect historical interactions between different *H. pylori* strain populations which promoted the inter-population exchange of particular hspAmerind *tfs3* alleles.

**Table 5:** Phylogeographic distribution of distinct alleles of the hypervariable subset of *tfs3* left flank genes

		Prevalence (%) of distinct alleles (1–5) of the four most variable *tfs3* left flank genes in *H. pylori* populations															
		t3_V24. (*pz*36)				t3_C3. (*pz*35)					t3_C2. (*pz*34)				t3_V25. (*pz*30)		
Population	Strains (*tfs3*+)^1^	1	2	3	4	1	2	3	4	5	1	2	3	4	1	2	3
hpEurope	34	25 (74)	1^a^ (3)	5 (15)	–	27^1^ (79)	1^a^ (3)	–	4 (12)	–	25 (74)	–	–	4 (12)	21 62)	1 (3)	4 (12)
hpAfrica1	53	45^d^ (85)	–	2^b^ (4)	4 (8)	45^d^ (85)	–	–	2 (4)	5 (9)	42^d^ (79)	–	5 (9)	2 (4)	43^c^ (81)	–	4 (8)
hpAfrica2	0	–	–	–	–	–	–	–	–	–	–	–	–	–	–	–	–
hpAsia2	5	–	3^a^ (60)	2 (40)	–	–	2 (40)	2^b^ (40)	–	–	1 (20)	3^a^ (60)	–	1 (20)	4 (80)	1 (20)	–
hspEAsia	14	–	12^d^ (86)	3 (21)	–	–	12^d^ (86)	2 (14)	–	–	–	11^d^ (79)	–	4^a^ (29)	2^a^ (14)	11^d^ (79)	–
hspAmerind	5	1 (20)	–	2^c^ (40)	2^a^ (40)	1^a^ (20)	–	–	2^a^ (40)	2^a^ (40)	–	–	2^a^ (40)	2 (40)	–	–	5^d^ (100)
Totals	111	71 (64)	16 (14)	14 (13)	6 (5)	73 (66)	15 (14)	4 (4)	8 (7)	7 (6)	68 (61)	14 (13)	7 (6)	13 (12)	70 (63)	13 (12)	13 (12)

^1^*tfs3*+ defined as ≥2 genes in the *tfs3* left flank. *P* value was determined by Fisher’s Exact Test and indicates significant association (positive or negative) of the indicated *tfs3* allele with a particular MLST. ^a^*P* < 0.05, ^b^*P* < 0.01, ^c^*P* < 0.001, ^d^*P* < 0.000, ^1^*P* = 0.052

### Heterogeneous distribution of the hspAmerind *tfs3* ICE

Since *tfs* ICEs are indicated to be mobile it is reasonable to consider that the heterogeneity of strain background in which hspAmerind *tfs3* left flank alleles are found could be readily explained by transfer of native hspAmerind ICEs to other phylogeographic *H. pylori* populations. To investigate this, nucleotide sequences of the four most variable *tfs3* genes t3_C3, C2, C5 and V25 from 75 strains were concatenated then used for construction of a Neighbour-Joining phylogenetic tree as before. Sequences were assigned the MLST population of the host strain and further annotated with an allelic profile derived from the collective Neighbour-joining analysis of the individual variable genes.

**Fig. 5:**
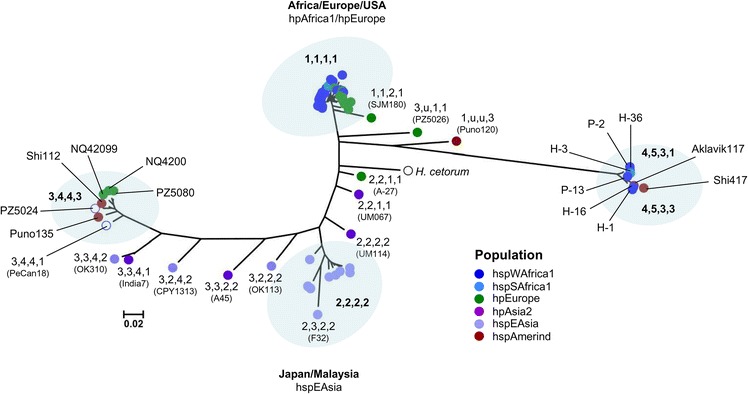
Neighbour-joining analysis of the *tfs3* variable gene subset. The phylogenetic tree was calculated from concatenated sequence of four *tfs3* variable subset genes comprising in order, t3_V24, t3_C3, t3_C2 and t3_V25 from 76 genomes. All positions in the initial sequence alignment containing gaps and missing data were eliminated prior to analysis. The four-digit number codes correspond to different alleles of each gene determined from previous phylogenetic analysis of individual genes (Additional file 3: Figure S5c, d). Two principal clades correspond to sequences from strains of hpAfrica1/hpEurope (clade 1) and hpEAsia (clade 2) lineages. The two remaining clades demonstrate characteristic high divergence of hspAmerind sequences but notably also include sequences from other *H. pylori* phylogeographic groups. A diversity of other unclustered allelic profiles arising from the inter-population exchange of variable subset *tfs3* genes or gene fragments are also apparent. Coloured circles representing gene sequences are coloured according to phylogeographic lineage of the originating strain as indicated. u; unassigned (phylogeny could not be determined due to substantially admixed sequence). Uncoloured circles indicate sequences from strains which could not be definitively assigned to a particular phylogeographic population

The majority of sequences could be identified within one of four major clades, two with homogeneous populations corresponding to either hpAfrica1/hpEurope (allelic profile, ‘1111’) or hspEAsia (allelic profile, ‘2222’) (Fig. 5 and Additional file 4: Table S4) and as before, two clades more heterogeneous in population structure (‘3443’ and ‘4533’) presumably corresponding to distinct hspAmerind populations. Consistent with this, multiple distinct subdivisions of hspAmerind populations have been resolved previously [17]. That the subset of variable genes that these ostensibly hspAmerind profiles represent appear conserved in other phylogeographic *H. pylori* populations suggests that the encoding left flank segment of the *tfs3* ICE was indeed acquired into these backgrounds as a single large block, most likely as a consequence of transfer of the entire *tfs3* ICE. A diversity of other unclustered allelic profiles representing unique combinations of variant alleles were also identified (Fig. 5). However, as these comprised more discrete substitutions of genetic material they might equally have resulted as a consequence of transformation and recombinational exchange of individual genes or gene fragments rather than ICE mobilisation.

To more fully resolve the genetic character of a selection of ICEs, we performed global pairwise sequence alignments, applying a stringent sequence identity cut-off (≥ 98%) to highlight distinct ICE regions homologous to each of three reference ICEs used as alignment scaffolds (hpAfrica1 or hspAmerind). These clearly showed admixture in the sequence of all hspAmerind-type *tfs3* ICEs resident in a heterologous strain background, suggesting some level of inter-ICE recombination prior to full or partial displacement of the native ICE-type (Additional file 3: Figure S6).

### Preservation of a *tfs3*-like ICE in other *Helicobacter* species

In addition to a variety of other species-specific clusters of T4SS genes, several other *Helicobacter* species harbor complete or remnant *H. pylori*-like *tfs* ICEs (Additional file 2). In particular, a large ca. 55 kb *tfs3*-like ICE is apparent in *H. cetorum* strain MIT-00-7128 [63], isolated from a captive Beluga whale, which, with the exception of a three gene insertion, is homologous to the corresponding *H. pylori tfs3* along its entire length (Fig. 4a). Strikingly, this homology also extends to the definition of an allelic profile from the subset of variable left segment genes, most closely resembling that of the admixed *H. pylori* sequence from strain A-27 (Fig. 5). The variable segment of the *tfs3* ICE therefore appears to have remained remarkably stable in these diverse backgrounds and seemingly subject to minimal host-adaptive changes. The prevalence and diversity of *tfs3*-like ICEs in the *H. cetorum* population is presently unknown, however, the presence of an *H. pylori*-homologous *tfs3* left segment cluster of genes (t3_C9-V3 inclusive) in a second *H. cetorum* strain, MIT 99–5656 [63], isolated from a wild dolphin, suggests that these ICEs may indeed be widespread and, by implication, long-associated with *H. cetorum* strains.

Apparent remnants of *tfs3* ICEs are also evident in two draft genomes of the porcine pathogen *H. suis* (Additional file 2). The *tfs3* fragment in *H. suis* strain HS1 [64] spans genes t3_C9 to t3_C10 inclusive on one WGS contig (00063), continuing to t3_C14 on a second (00062), although several large deletions are apparent relative to *H. pylori tfs3* ICEs, conceivably consequent with adaptive changes following initial acquisition [18]. However, whereas the *H. cetorum tfs3* is somewhat sequence divergent from *H. pylori tfs3*, consistent with the sequence divergence of both genomes in general [63] (and also indicated by moderate sequence similarity of concatenated MLST sequences, Additional file 4: Table S5), the *H. suis tfs3* sequences, in marked contrast, remain highly similar to the *H. pylori tfs3* ICE, demonstrating 91-98% identity over the three gene clusters compared and ≥98% sequence identity over much of the t3_C9 to t3_C10 fragment when directly compared with the equivalent *tfs3* segment from hpAfrica1 *H. pylori* strain Gambia94/24 (Additional file 3: Figure S7). This is particularly surprising given the substantial separation of *H. suis* and *H. pylori* species (68% identity between MLST concatenated sequence, Additional file 4: Table S5) and suggests a recent acquisition of *H. pylori tfs3* by the *H. suis* HS1 strain.

## Discussion

Difficulties in obtaining contextual information for large contiguous clusters of genes from draft genome sequences has undoubtedly presented a bottleneck in the study of large genomic elements such as ICEs, not least the *tfs* ICEs of *H. pylori*. In addressing this issue we have significantly extended observations from analysis of limited numbers of complete genome sequences, enabling detailed assessment of *tfs* ICE structure, variation and prevalence within the global *H. pylori* population. From this foundation, we have provided a novel classification of the *tfs* ICEs which has facilitated study of their historical association and mobility within and between geographically diverse human populations.

### Distribution and distinctive variation of the *tfs* ICEs

*H. pylori* is considered to have infected anatomically modern humans since their emergence in Africa ~100kya, continuing to co-evolve with different human populations following their migratory expansion from Africa, beginning ~60kya [13, 14, 16]. Genetically distinct populations of *H. pylori* can be discerned as a consequence which reflect human colonisation of particular geographic regions and their subsequent interactions in both pre- and contemporary history. Currently, seven main *H. pylori* lineages are apparent, of which hpAfrica2 is considered to be ancestral, predating human migration out of Africa [13, 14, 16]. The ubiquitous presence of the *tfs4* ICE in all phylogeographic *H. pylori* populations, including hpAfrica2 is therefore particularly striking, and alludes to the association of *tfs4*, and more particularly the L1C1R1 subtype, with an ancestral population of *H. pylori* potentially before spatial separation. That a fragment of the *tfs4* C1 module is also apparent in *H. acinonychis*, which is part of the same super-lineage as hpAfrica2 [18] supports such an ancient origin.

In contrast, the lower abundance and population skew of L2C2R2 subtype modules suggests a different evolutionary history, perhaps reflecting later acquisition of this variant and/or adaptation to particular isolated *H. pylori* populations. With the exception of the notable predominance of the L2C1R1f *tfs4* type in hpAfrica1 strains, L2/R2 flanks are otherwise most frequently associated with hpEAsia/hpEurope strains (Table 3) suggesting a possible ancestral association with early human inhabitants of the Eurasian continental landmass. Subsequent interactions between both human and *H. pylori* strain populations, enabling hybridisation of L1C1R1 and L2C2R2 subtypes, might then account for the introduction and stable inheritance of the L2 module within the hpAfrica1 strain population in the form of the transfer-deficient L2C1R1f hybrid. Although speculative, this general model is consistent with theories of human migrations out of Africa, in which waves of migration from North East Africa and Central Asia are considered to have converged in Western Asia 10-52kya, followed by human expansion throughout Europe, and ultimately, back migration to Africa from Europe and the Middle East ~10kya [16, 19].

Although apparent in the majority of *H. pylori* populations, the absence of the *tfs3* ICE in hpAfrica2 strains, and indeed one third of all strains in this study (Figs. 1 and 3c, Table 4), is also suggestive of its acquisition contemporary to L1C1R1 *tfs4*. However, the clear association of individual *tfs3* alleles with particular *H. pylori* lineages similarly alludes to significant co-evolution with geographically diverse *H. pylori*-infected human populations. Phylogeographic signals are particularly apparent for the subset of variable genes encoded within the left flank of *tfs3*, for which multiple distinct allelic forms can be discerned. Although the *tfs3* variable subset comprises several genes unique to the *tfs3* ICE, homologues of two, t3_c2 and t3_C3, are also apparent in both *tfs4* ICE subtypes as one of two stably conserved, albeit highly divergent variants (Additional file 3: Figure S5). The reason for the difference in patterns of variation between these latter *tfs* genes is unclear, although it might presumably reflect the role of the encoded proteins relative to the overall function of each ICE within particular strain-host populations.

### Functional considerations of modules and hybrid L-C-R modular configurations

ICEs are common in many bacterial species, invariably persisting within populations as a consequence of a particular fitness advantage they confer upon the host strain [65]. A modular structure is common, with particular modules conferring functions relevant to different ICE activities, including conjugation (encoding mating-pair formation [MPF] T4SS genes), transfer (encoding VirD2 relaxase and VirD4 coupling protein) and recombination (encoding relaxase and integrase/excisionase functions) [65]. These and other modules typically also harbour the adaptation/accessory genes that contribute to the evolutionary success of both ICE and host strain. A modular disposition of the *tfs* ICEs is also clearly apparent, most notably for *tfs4* (Fig. 2) in which the C and R modules encode readily identifiable MPF and transfer functions [45], and the L modules a *xer*-mediated recombination function [44]. Common to other ICEs [66, 67], *tfs4* L modules also include a putative VirB6–homologous MPF protein encoded independently from the main *vir* gene complement. All three L-C-R modules are therefore presumably required for intercellular transfer competence as they all encode functions relevant to this role.

Although module boundaries are less distinct, *tfs3* in contrast appears to comprise two modules, broadly dividing the *tfs3* ICE into left and right sections which harbour either recombination (plus VirD2) or MPF functions (plus VirD4) respectively (Fig. 4a). Both *tfs4* and *tfs3* ICEs commonly demonstrate loss and fragmentation of MPF modules and more frequent preservation of substantially intact L modules (Figs. 1 and 3a and b, Additional file 3: Figures S2-S4, Tables 3 and 4). However, mutational inactivation and erosion of mobility modules is common in ICEs that confer an adaptive advantage [65] suggesting that the selective maintenance of *tfs* L modules in the great majority of *H. pylori* strains might be attributable to a particular benefit their encoded protein products confer independently of the rest of the *tfs* ICE, including the encoded T4SS. It can therefore be considered that *tfs* L-C-R modules encode for different activities which are both dependent (ICE mobilisation) and independent (undetermined functions) of each other. This notion is further strongly supported by the high prevalence of the mobility-defective L2C1R1f *tfs4* type (lacking essential *virB4, B3, B2* T4SS assembly genes in the truncated R1 module) in the hpAfrica1 strain population, and also provides an explanation for the occasional stable generation of diverse hybrid *tfs4* L-C-R modular configurations that may similarly be defective for T4SS assembly. With respect to the latter, it is unclear whether T4SS proteins encoded by orthologous, yet highly sequence divergent *tfs4* gene subsets contained within the different modules (Additional file 4: Tables S1 and S3) have the capacity for cross-complementation in the assembly of a functional hybrid T4SS. Of the ten *vir*-homologous T4SS assembly proteins that are encoded by L-C-R modules, only one, VirB9, is highly conserved between both L1C1R1 and L2C2R2 *tfs4* types (> 90% amino acid sequence identity, Additional file 4: Table S3). VirB9 is a core component in the T4SS assembly pathway, mediating interactions with multiple other Vir proteins in the formation of a stable secretion system complex [68]. It can therefore be assumed that there is at least some flexibility in the interactions of the conserved *tfs4* VirB9 protein with both sets of orthologous Vir assembly proteins. Whether this is similarly the case for the other Vir proteins for which two distinct variants are apparent (43-80% sequence identity, Additional file 4: Table S4) remains to be determined, although if such heterologous interactions are not permissible then hybrid ICEs may also be defective for T4SS activity.

The potential for functional complementation by homologous components of *tfs3* and *tfs4* ICEs, and indeed *tfs*-homologous proteins encoded elsewhere in the genome (Additional file 2 and Additional file 4: Table S1b) similarly remains to be established. However, as ICE MPF functions are often exploited for the mobilisation of other unrelated genomic elements [65], it is reasonable to speculate that the *tfs* MPFs could at least each function in the mobilisation of any other ICE (and possibly also discrete ICE modules) that retains transfer and recombination activity.

### Inter-population *tfs* ICE transfer and exchange

In support of previous observations from a more limited set of 36 CG sequences [44], Neighbour-joining analysis of *tfs* genes was frequently discordant with the distinct population divisions resolved by MLST, with predominant *tfs* clades more often comprising coalesced clusters of hpAfrica1/hpEurope (*tfs4* L2 modules and *tfs3* LF variable ‘1’ alleles) and hpAsia2/hspEAsia (*tfs3* LF variable ‘2’ alleles) populations (Fig. 5, Additional file 3: Figure S5). The lack of fine population resolution for these alleles suggests a different evolutionary history relative to the core genome of the host strain which can be readily explained by ICE mobility, involving perhaps frequent inter-population transfer and exchange of *tfs* ICEs or component modules and a mechanism of replication-independent site-specific recombination mediating ICE excision and integration [69]. This contrasts with the more congruent phylogeny of the *cag*PAI with the core genome [70] which is indicative of its immobility within *H. pylori* genomes possibly from the time of its initial acquisition.

From the available data, it appears that *tfs* transfers occur preferentially between particular strain populations (such as hpAfrica and hpEurope) which may reflect either a history of extended interaction and population admixture or host-specific adaptations which potentially restrict the population range of particular ICE/module/allele types. Indeed, given the lack of a distinct hpEurope clade for any of the studied *tfs* genes/alleles and the common occurrence of hpEurope strains within clades predominated by other populations (hpAfrica1 and hspAmerind) it could be speculated that *tfs*’s do not have an appreciable evolutionary history with hpEurope populations, but rather, that these strains have more commonly been recipient to ICEs from other *H. pylori* lineages. The hpEurope population evolved through gradual admixture of two ancestral *H. pylori* populations, ancestral Europe 1 (AE1) and ancestral Europe 2 (AE2), following human migrations from North East Africa (AE2) and Central Asia (AE1) 10-52kya [13, 14, 16]. However, recent evidence suggests that the significant AE1/AE2 admixture characteristic of modern hpEurope only occurred after the Copper Age, ~ 5-6kya [71]. Association of *tfs*’s with modern hpEurope strains may therefore be much more recent than for other populations in which they have co-evolved over more significant periods of time, possibly accounting for the increased pseudogenisation of ICEs observed in these strains (Fig. 1b).

Our analyses of *tfs3* ICEs in particular also provided evidence for inter-population transfer of hspAmerind ICEs to hpEurope and hpAfrica1 strains (Fig. 5, Additional file 3: Figures S5 and S6). No reciprocal transfers were observed suggesting that *tfs* transfers between these populations may be biased towards export rather than import, consistent with observations for DNA exchange within hspAmerind genomes as a whole [17]. In this context, hspAmerind strains have been shown to inefficiently transform with DNA from other *H. pylori* populations [30], potentially as a consequence of several strain-specific and functionally distinct restriction modification systems [72] which would similarly restrict the acquisition of foreign *tfs* ICEs. Such mechanisms may contribute to the low genetic diversity of hspAmerind strains and their consequent competitive displacement by other *H. pylori* populations [29, 30]. It is particularly noteworthy therefore that whereas hspAmerind strains may decline due to reduced fitness, their *tfs* ICEs by contrast, appear to have the capacity to survive the competitive interaction of mixed/transient infection to substantially displace the resident *tfs* ICE of competing foreign strains (Fig. 5, Additional file 3: Figure S6).

### Disease implications of *tfs* ICE carriage

Component marker genes of both *tfs* ICEs have been found to associate with increased risk for *H. pylori*-related disease in some populations, indicating that the ICEs may confer as yet undefined virulence functions [40, 51, 52, 54, 55]. Such associations generally relate to genes encoded by *tfs4* R1 (*dupA*+), L1 (*jhp0947*, *jhp0949*) or both L1/L2 (*jhp0945*) modules and the variably-encoded *tfs3* left module *ctkA* gene (*jhp0940*). However, it is unclear from these studies whether these associations, particularly in the context of *tfs4*, relate to the activity of individual proteins, particular modules, *tfs*-types or are dependent or otherwise upon the function of the ICE-encoded T4SSs. In these contexts, our observations that 1) *tfs4* L and R modules occur with high frequency in the global population, often in isolation from other ICE modules, and 2) the immobile L2C1R1f-type remains highly conserved without significant erosion in hpAfrica1 strains, both suggest that the L1/L2 modules may confer important function independently of Tfs4 T4SS activity. With respect to the latter, it is noteworthy that a recent study reported a remarkably low incidence of *H. pylori*-related disease in a Nigerian population infected with strains with the L2C1R1f *tfs4*-type but an otherwise highly virulent *cagA*/*vacA* genotype [27]. As the full R1 module (*dupA*+) is invariably associated with a C1 module (T4SS+) and defines an increased risk for duodenal ulcer and possibly reduced risk of gastric cancer [48], it is intriguing to speculate that in the absence of presumptive T4SS function, the L2C1R1f-type (or L2 module alone) might conceivably have a protective role in some settings; interactions of *H. pylori* with its human host which have a beneficial rather than pathogenic outcome have been suggested previously [9, 20, 22]. At the least, it can be considered that the prominence of the L2C1R1f *tfs4*-type in African strains, either alone or in addition to other factors, such as a concomitantly low co-occurrence of *tfs3* (Fig. 3c), might contribute to overall reduced pathogenicity. With regard to the latter, as certain *tfs3* genes have been reported to associate with increased risk of gastric cancer [40, 52, 55, 56], the low co-occurrence of T4SS-competent *tfs3* and L1C1R1 *tfs4* (Fig. 3c) might similarly be proposed as a factor in the observed inverse association of *dupA+ H. pylori* with this particular outcome of *H. pylori* infection [48].

In additional consideration of *dupA*, we also note that 29% of R1 (*dupA*+) modules are apparent in strains lacking a complete L1C1R1 *tfs4* (Fig. 3b and c) suggesting that *dupA* presence alone is not a reliable marker for an intact *tfs4* ICE and the functions it encodes.

### *tfs* ICE transfer between populations and species

Whereas mutualistic interaction within co-evolved *H. pylori*-human populations is suggested to limit pathogenicity, discordant bacteria-host ancestry in contrast has been proposed to increase the risk of gastric disease [22, 26]. As *tfs* ICEs show evidence of evolution within particular strain populations (hpAfrica1/hspEAsia/hspAmerind in particular) it could be considered that inter-population heterologous exchange of ICEs (Fig. 5) might also change the virulence character of otherwise native host strains, possibly with detrimental consequences to the human host.

In addition to *tfs* ICE mobilisation between different *H. pylori* populations, the remarkable sequence similarity of the left segment remnant *tfs3* ICE from the porcine *H. suis* strain HS1 with *H. pylori tfs3* (Additional file 3: Figure S7) is intriguing and suggests contemporary interaction and ICE exchange between different *Helicobacter* species. *H. suis* is a recognised zoonoses and the most prevalent gastric non-*H. pylori Helicobacter* capable of causing gastric disease in humans [2, 73, 74]. Although the route of *H. suis* transmission from swine to humans is unresolved, transient co-colonisation of the human gastric niche with both *H. suis* and *H. pylori* would present reasonable opportunity for *tfs* ICE exchange. However, the isolation of the *H. suis* HS1 strain from the gastric mucosa of an infected swine [64] invokes more speculative models of *tfs3* ICE acquisition, requiring either transient infection of the source animal with *H. pylori*, or human to animal (re)transmission of the HS1 strain. These models of anthroponotic infection remain unexplored, although are plausible as *H. pylori* has proven capacity to infect other animal species [75, 76] and is the likely ancestral origin of *H. acinonychis* [18]. Important clarity is therefore required regarding contemporary *tfs3* gene flow between *H. pylori* and *H. suis*, since a human adapted *H. pylori tfs3* could conceivably confer attributes which influence colonisation and/or virulence by *H. suis* within a human or porcine host.

## Conclusions

In conclusion, the *tfs* ICE environment of individual *H. pylori* strains is shown to be complex and highly variable, reflecting both ancient and contemporary accretion, erosion and exchange of different ICE types and their genetically distinct component modules. That *tfs* modules might encode for activities which are both dependent and independent of each other suggests that inclusion of full ICE modular representation in the definition of a strain *tfs* genotype will aid understanding of the function and disease risk potential of particular ICE modular types. Finally, further knowledge of *tfs* gene flow within and between different *Helicobacter* populations and species will provide important context in assessing the beneficial or detrimental impact of different ICE types on both strain fitness and health of the infected human host.

## Methods

### Bioinformatics analyses and generation of datasets

*tfs* ICE gene clusters were identified within complete *H. pylori* genome sequences obtained from the NCBI [77, 78] or PATRIC [79, 80]. Sequences were compared in pairwise and multiple sequence alignments as appropriate (Needle/Stretcher and ClustalOmega/KAlign [81] respectively, accessible from [82]), then manually re-annotated to establish precise definition of coding sequence and full gene content using BioEdit [83, 84] and the suite of analysis tools available through the EXPASY resource [85, 86]. This comprehensive analysis resolved disparities arising from automated database annotation of frameshifted genes (not annotated or annotated as multiple individual genes) and provided clear discrimination of variant alleles.

Reference sequences selected from these comparative analyses, representative of the entire *tfs* gene pool (59 genes) were translated and subsequently used in sequential BLASTp (NCBI BLAST version 2.2.22, BLOSUM62 matrix) [77] interrogation of the RefSeqProt database (contemporary to June 2015) within the PATRIC bioinformatics platform [79] to obtain *tfs* homologous sequences from available draft WGS sequences. Significant hits were collated into an ordered searchable dataset comprising ~ 56,000 nucleotide and amino acid sequences (Additional file 1). The latter were initially identified and broadly tagged as either *tfs3* or *tfs4* ICE-encoded components by the presence of conserved sequence motifs characteristic of each (determined from previous multiple sequence alignment of reference sequences). This facilitated rapid sequential determination of full *tfs* gene representation and context relative to intact reference *tfs3* and *tfs4* ICEs (from strains PeCan18 and G27/P12 respectively) for each of 221 *H. pylori* genomes, comprising 53 complete (including 8 pairs/replicates) and 168 WGS (including 26 pairs/replicates) genome sequences (Additional file 2). Representation of homologous genes separate from the *tfs* ICEs and those present in 11 non-*pylori Helicobacters* were also included in the dataset in addition to the complement of *com* genes and a subset of nine *cag* genes selected at intervals along the entire length of the *cag*PAI (ca. 30 kbp). These latter were included to assess the utility of the overall approach for effective determination of complete gene subsets which may be encoded in both multiple separate genomic locations (*com*, akin to fragmented *tfs* ICEs) or in a single large contiguous cluster (*cag*PAI comprising 28–30 conserved genes, akin to complete intact *tfs* ICEs). Selected subsets for both *com* and *cag* genes were identified in their entirety for the majority of strains with isolated exceptions due to bona fide deletion of the complete *cag*PAI/individual genes or lack of automated database annotation for specific genes due to multiple frameshift mutation (Additional file 2). This was similarly found to be the case for *tfs3* and *tfs4* ICEs following validation of the complements of BLASTp *tfs* hits against NCBI database entries of complete genomes and cross-reference to the initial manual re-annotation of the *tfs* clusters.

Percentage sequence identity matrices for selected genes were generated from multiple sequence alignments in ClustalOmega [82]. Allelic variants with sequence identity < 70% were subsequently assigned to an arbitrarily numbered allelic group on the basis of evolutionary distance inferred using the Neighbour-joining method in MEGA 6.0 [87] as described below. Representation and definition of *tfs* ICE modules and subtypes was deduced from contiguous gene content and clusters annotated accordingly for all strains. A facile nomenclature based on *tfs4* gene order and accounting for all conserved and variable *tfs*-homologous genes was additionally developed for standardised description and ease of reference to genes in the final datafile (Additional file 2). Additional meta-data for individual *H. pylori* strains was obtained from a survey of genome sequence entries in Genbank [78] and associated publications.

### MLST and phylogenetic analyses

All *H. pylori* strains interrogated for *tfs* gene content were assigned to previously established populations or subpopulations [13] using multilocus sequence typing (MLST). Briefly, partial sequence of seven housekeeping genes, *atpA*, *efp*, *mutY*, *ppa*, *trpC*, *ureI* and *yphC* for each strain was obtained as described above, concatenated, then sequences aligned together with a selection of 347 reference sequences obtained from the MLST database [88, 89] using the MUSCLE alignment program in MEGA 6.0 [87]. Alignments were subsequently used for generation of phylogenetic trees in MEGA 6.0 using the Neighbour-joining method and Kimura 2-parameter model of nucleotide substitution with 1000 bootstrap replicates (Additional file 4: Figure S1). Evolutionary relationships of both individual and concatenated sequence derived from a subset of variable *tfs3* genes from 76 complete and draft genomes (Additional file 5) was inferred using a similar approach. All positions in the alignment containing gaps and missing data were eliminated prior to analysis leaving 2474 positions in the final dataset. Phylogenetic trees were also constructed for individual *tfs* genes using CLUSTALW codon alignments, but otherwise identical parameters in MEGA 6.0.

### Comparative alignment of *tfs* ICE sequences

Comparative analyses of selected contiguous *tfs* ICE sequences contained within individual FASTA sequence files was done in mVISTA using the Shuffle-LAGAN alignment program [90]. Similarity between aligned sequences was depicted using a 50 bp calculation window and sequence identity cut-off ranging from 70 to 98%.

### Analyses in the R programming environment

To illustrate the representation of *tfs* and *cag* genes in *Helicobacter* complete and draft genomes, gene content matrices were built in the R (v3.2.2) programming environment [91, 92] using the ‘gplots’ package [93]. Loci were hierarchical clustered by similarity of content using the ‘hclust’ function (ward.D2 method and Euclidean distance measure) to generate a sidelong dendrogram. The R environment was also used for representation of *tfs* and *cag* gene co-occurrence in relevant *H. pylori* genomes using the VennDiagram package [94].

### Statistical analyses

Fisher’s Exact test was used for statistical analysis of contingency tables using Graphpad Prism 7.01 (GraphPad Software, California, USA).

## Additional files

Additional file 1: Dataset 1. This file contains compiled BLASTp search results for each of 59 *tfs*-encoded proteins. (XLSX 3851 kb)

Additional file 2: Dataset 2. This file contains complete datasets for *tfs*, *com* and (partial) *cag* gene content extracted from 232 *Helicobacter* strains, additionally including gene/*tfs* ICE description, classification and nomenclature and relevant strain meta-data. (XLSX 249 kb)

Additional file 3: Figures S1 – S7. This file contains **Figures S1** – **S7** and associated Figure legends. (PDF 9338 kb)

Additional file 4: Tables S1 – S5. This file contains **Tables S1** – **S5**. (PDF 52 kb)

Additional file 5: Dataset 3. This file contains FASTA-formatted concatenated sequence of *tfs3* ICE genes t3_V24, t3_C3, t3_C2 and t3_V25 from 76 *H. pylori* and *H. cetorum* genomes. (XLSX 50 kb)

Additional file 6: High quality, 600dpi resolution version of Fig. 1. (TIF 14438 kb)

## Abbreviations

ICE: Integrative and conjugative elements
MLST: Multi locus sequence typing
PAI: Pathogenicity island
T4SS: Type IV secretion system
WGS: Whole genome shotgun (sequence)
