# Supplementary material for: Phylogeographic diversity and mosaicism of the Helicobacter pylori tfs integrative and conjugative elements
Source: Mob DNA. 2018 Jan 26;9:5. doi: 10.1186/s13100-018-0109-4 (PMC5785829; doi:10.1186/s13100-018-0109-4)
Supplement: Supplementary file 3 — This file contains Figures S1 – S7 and associated Figure legends. (PDF 9338 kb) [file 13100_2018_109_MOESM3_ESM.pdf]

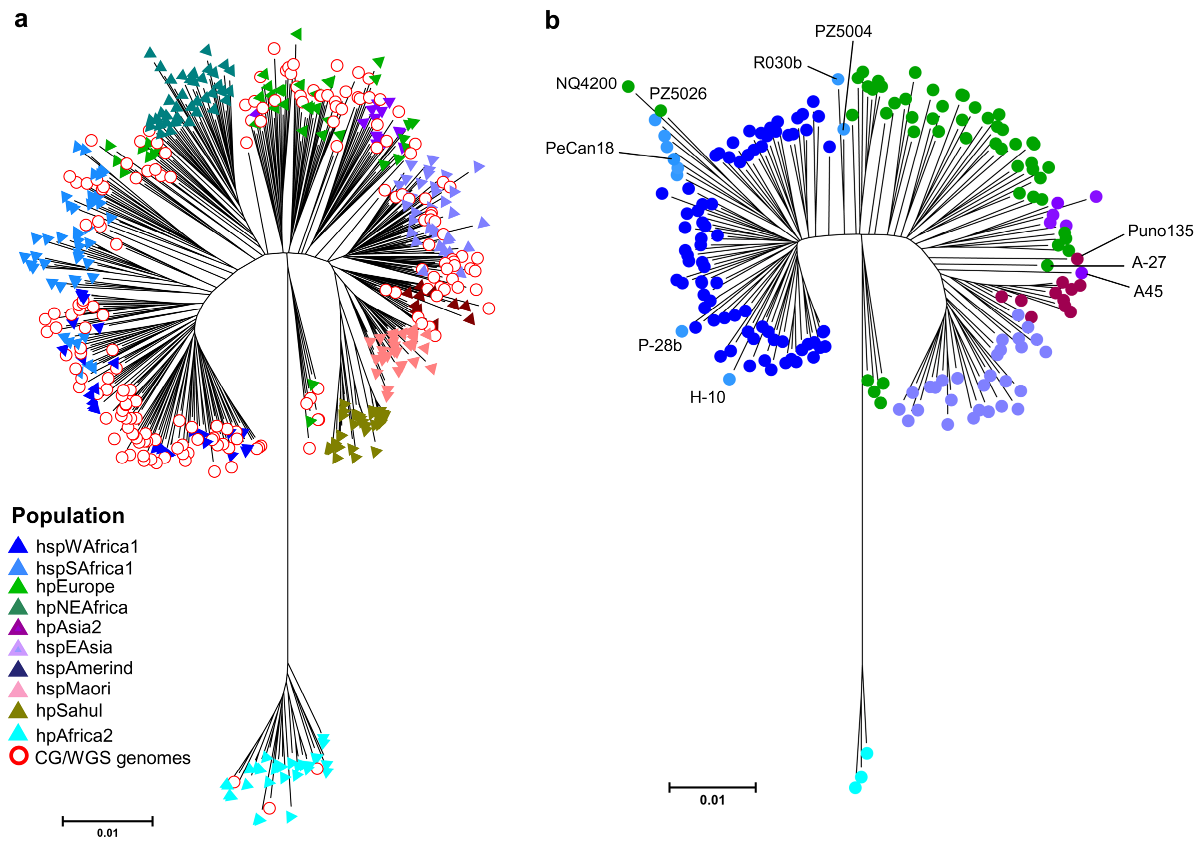

Fig. S1. Phylogeographic diversity of *H. pylori* strains. a. The Neighbour-joining phylogenetic tree was calculated from concatenated sequence of seven housekeeping genes from all genomes interrogated for *tfs* ICE content in addition to 347 reference sequences obtained from the *H. pylori* MLST database. Coloured triangles represent different established phylogeographic lineages of *H. pylori* as indicated. Open red circles indicate phylogeographic lineage of complete (CG) and draft (WGS) genome sequences used in this study. **b.** Neighbour-joining tree calculated from concatenated sequences of CG and WGS genomes only to confirm phylogeographic assignments established in a. Strain genomes with ambiguous assignments are labelled. Phylogeographic assignments for all strains are listed in Additional file 2.

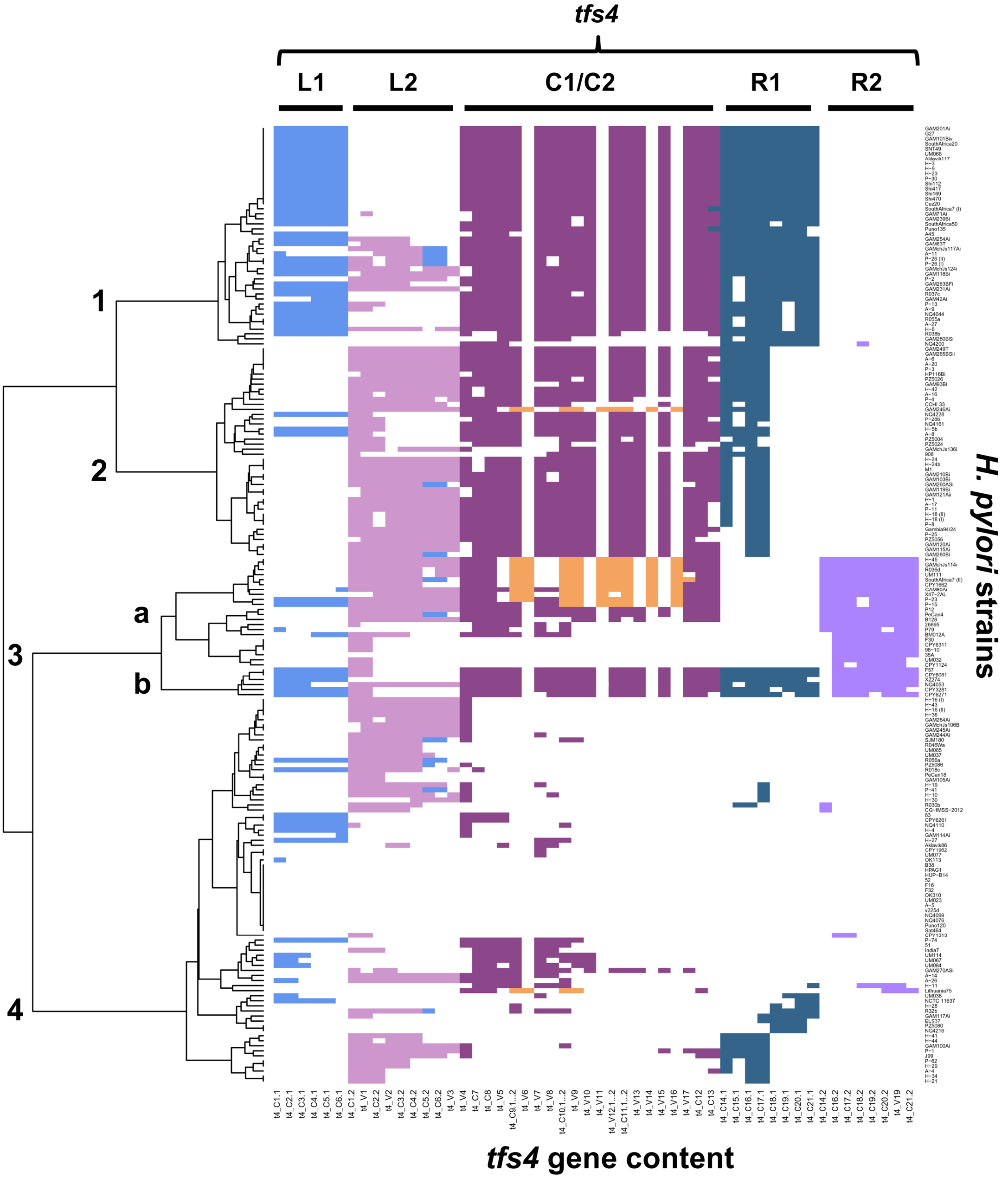

Fig. S2. Prevalence and status of the *tfs4* ICE in 187 *H. pylori* genomes. Colouring of *tfs4* gene subsets corresponds to different *tfs4* ICE modules. Hierarchical clustering broadly delineates different *tfs4* ICE types present as substantially intact clusters in ~50% of the global strain population (cluster 1, 2 & 3b). Remaining strains with remnant ICEs are often seen to retain a complete left (cluster 4) or right flank (cluster 3a) module complement of genes.

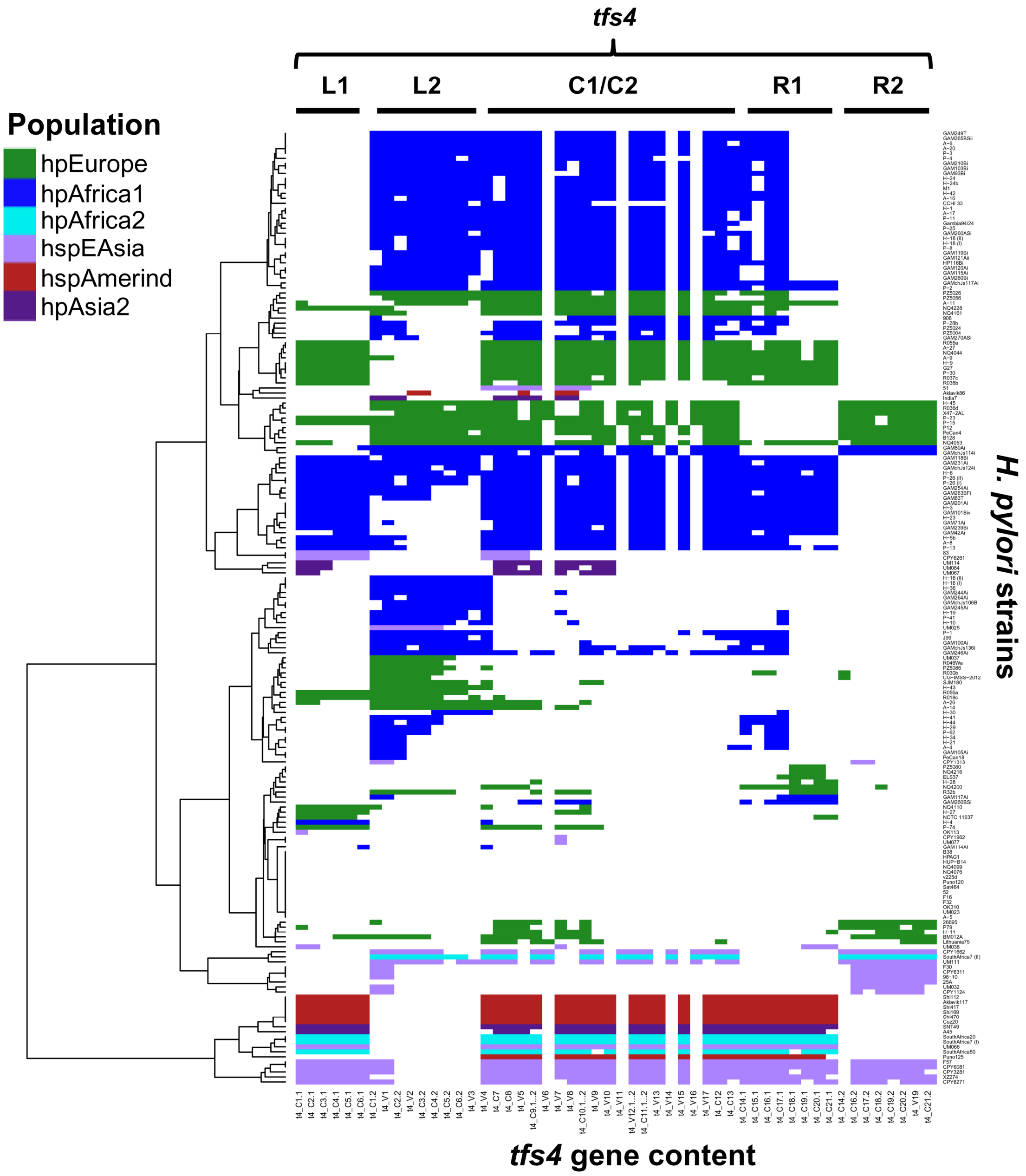

Fig. S3. Phylogeographic distribution and status of the *tfs4* ICE in 187 *H. pylori* genomes. *tfs4* gene content data transformed to show distribution of *tfs4* type and representation in different phylogeographic populations.

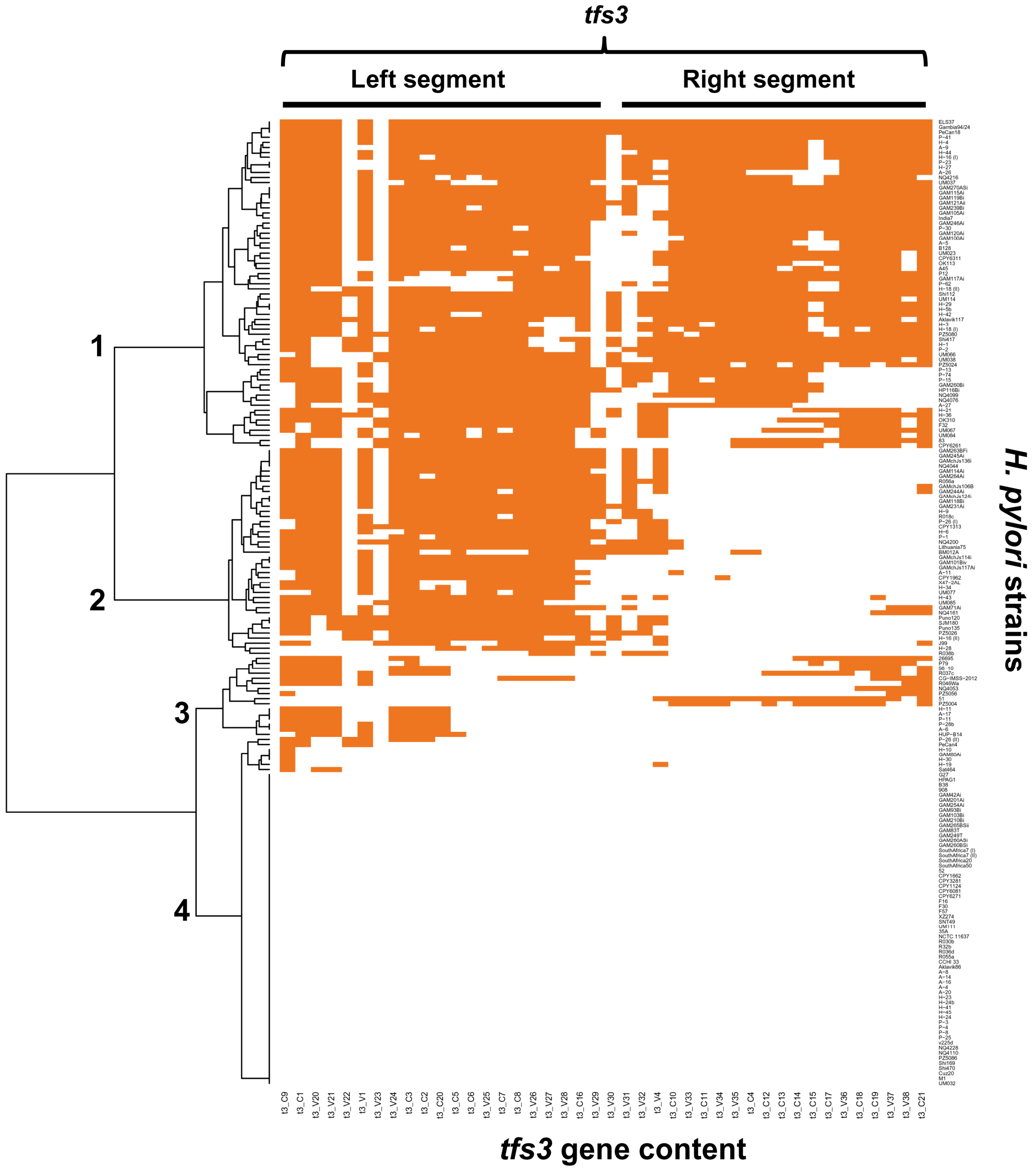

Fig. S4. Prevalence and status of the *tfs3* ICE in 187 *H. pylori* sequenced genomes. The left segment of the *tfs3* ICE is identified in ~50% of strains (clusters 1 and 2), approximately half of which also encode a contiguous right segment as a substantially intact *tfs3* ICE (cluster 1). The majority of remaining strains lack any evidence of previous carriage of a *tfs3* ICE (cluster 4).

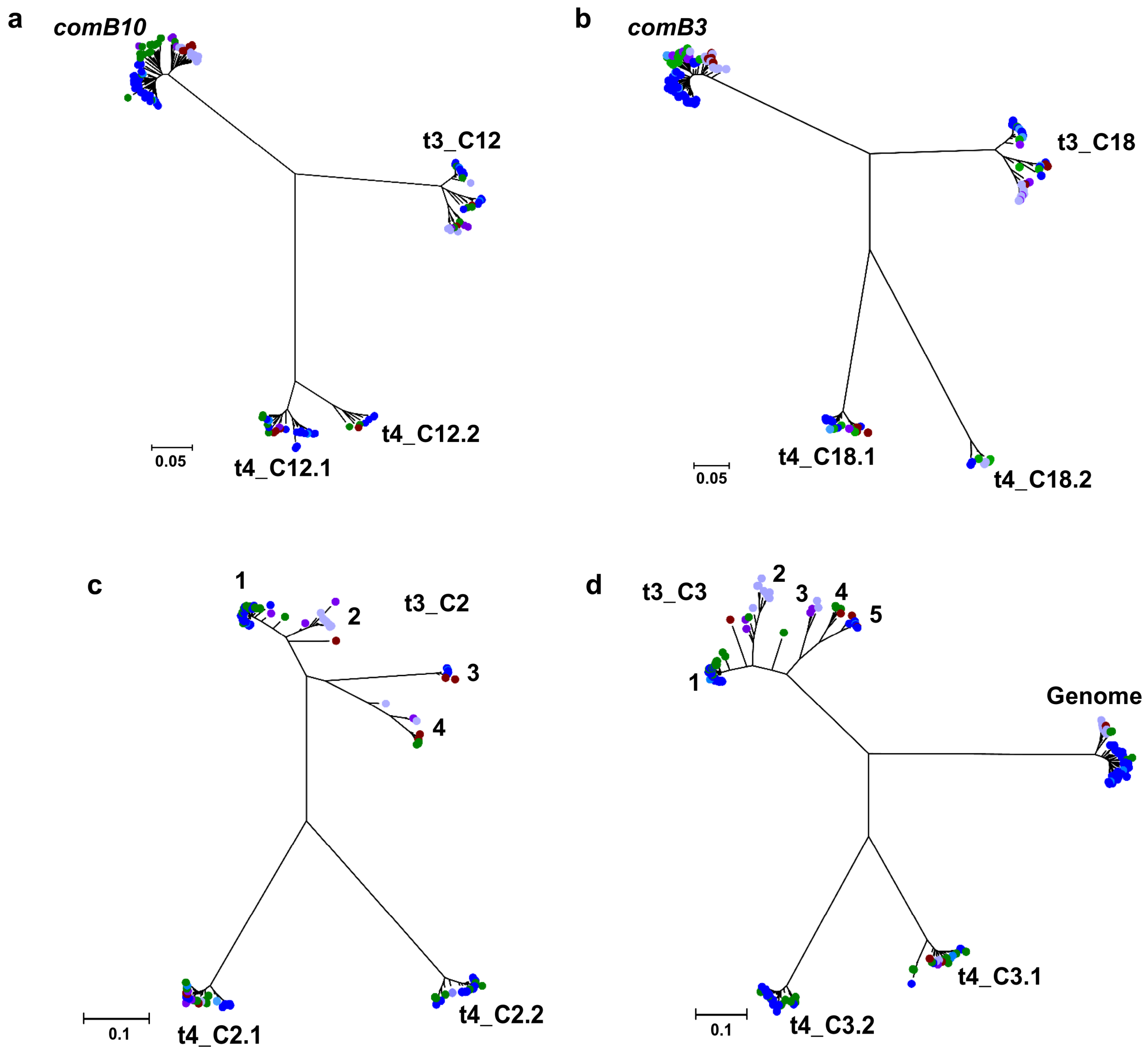

Fig. S5. Neighbour-joining phylogenetic tree of representative conserved and variable *tfs* ICE genes and sequence homologs. Genes were selected to represent the extent of allelic variation within the three different central (panel a [C12/*virB10*]), right (panel b [C18/*virB3*]) and left (panels c [C2/*virB6*] and d [C3]) *tfs* ICE modules. Two well conserved but highly divergent allelic variants are apparent for the majority of *tfs4* genes, corresponding to each of the two principal *tfs4* ICE types. *tfs3* variation in contrast is mainly restricted to a hyper-variable subset of left segment genes. As illustrated for two genes of the *tfs3* variable subset (panels c and d), up to five allelic variants can be discerned for each gene in addition to multiple other admixed sequences. Whereas phylogeny of *tfs* genes is generally incongruous with that of the host strain genome, two principal clades can be identified which broadly comprise sequences from strains of hpAfrica1/hpEurope (clade 1) and hpEAsia (clade 2) lineages. The remaining clades are more mixed but characteristically comprise hspAmerind gene sequences. Coloured circles representing gene sequences are coloured according to phylogeographic lineage of the originating strain as indicated in Figure S1.

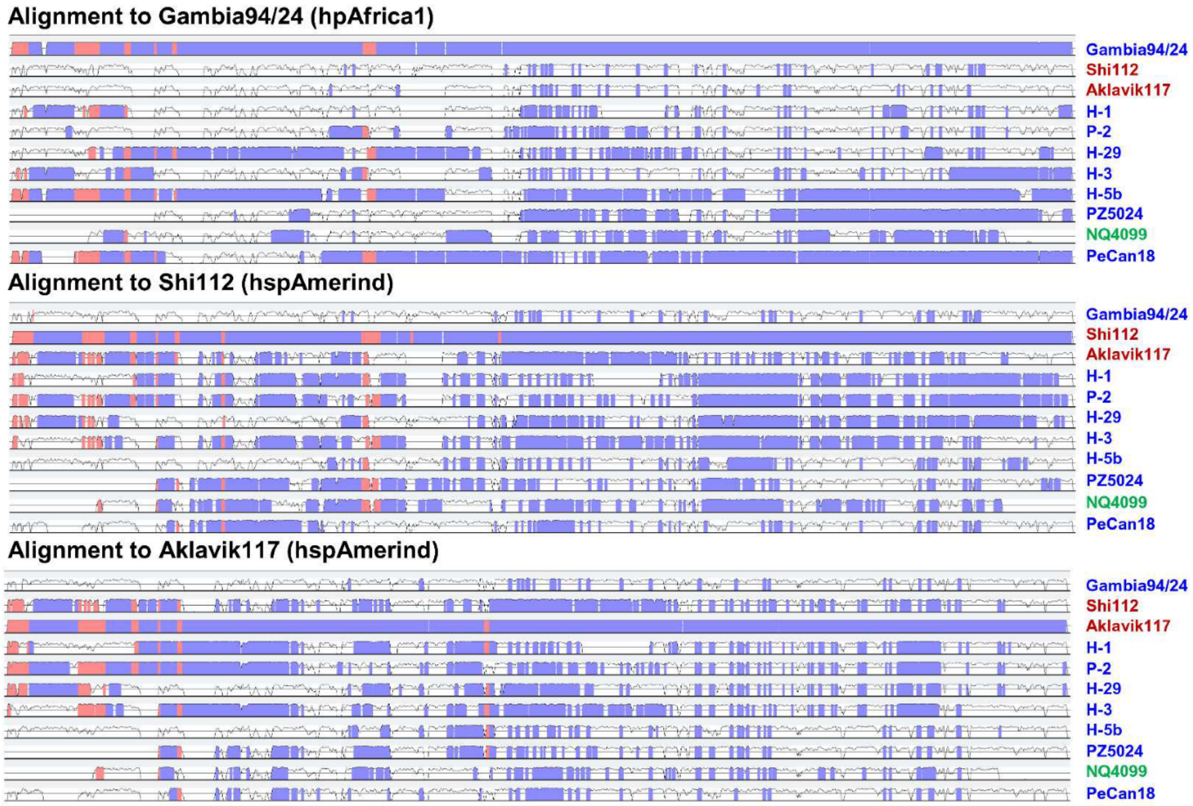

Fig. S6. Inter-lineage transfer and mosaicism of hspAmerind *tfs3* ICES. Global pairwise sequence alignments of different *tfs3* ICEs against each of three reference *tfs3* ICE sequences (from strains Gambia94/24, Shi112 and Aklavik117) was performed using the Shuffle-LAGAN alignment program in mVISTA. Individual mVISTA graphs depict sequence similarity of *tfs3* ICEs from the indicated strains relative to the indicated reference *tfs3* ICE using a 100bp calculation window and 98% sequence identity cut-off. The mid-line in individual alignment graphs corresponds to 50% sequence identity to the Gambia94/24 reference *tfs3* ICE sequence and regions with sequence identity ≥98% are indicated by blue (coding sequence) or red (non-coding intergenic sequence) colouring. Gaps indicate absent genes. Coloured labelling of strains corresponds to MLST population as follows; blue/hpAfrica1, red/hspAmerind, green/hpEurope. Individual graphs reveal evidence for interlineage transfer of hspAmerind *tfs3* ICEs with subsequent admixture or displacement of the resident native ICE.

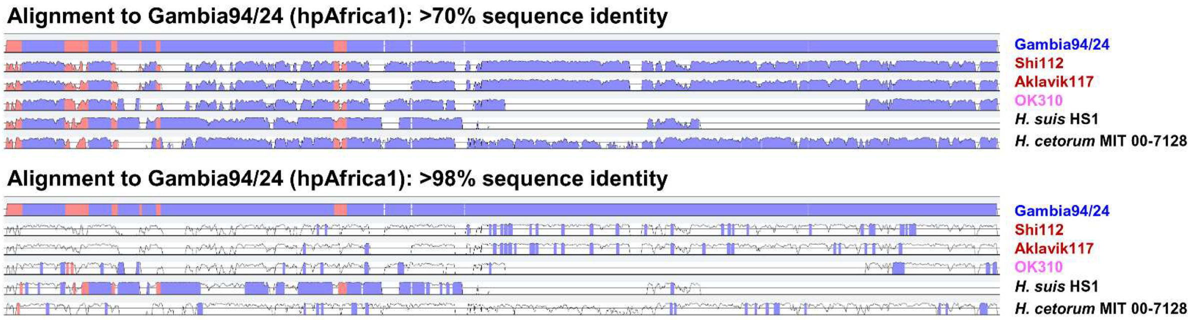

Fig. S7. Sequence similarity of the *H. suis tfs3* ICE remnant with *H. pylori* hpAfrica1 *tfs3*. The full *tfs3* ICE sequence from hspWAfrica strain Gambia94/24 was used as reference scaffold for global pairwise sequence alignments of different *tfs3* ICEs in mVISTA using a 100bp calculation window. The mid-line in individual alignment graphs corresponds to 50% sequence identity to the Gambia94/24 reference *tfs3* ICE sequence and regions with sequence identity ≥70% (top panel) or ≥98% (bottom panel) are indicated by blue (coding sequence) or red (non-coding intergenic sequence) colouring. Gaps indicate absent genes. Coloured labelling of strains corresponds to MLST population as follows; blue/hspWAfrica, red/hspAmerind, pink/hspEAsia. The *H. suis* remnant *tfs3* ICE comprising the left segment of variable genes, is shown to be almost identical to the corresponding region of the Gambia94/24 *tfs3* ICE over most of its sequence.
