# Supplementary material for: Phylogeographic diversity and mosaicism of the Helicobacter pylori tfs integrative and conjugative elements
Source: Mob DNA. 2018 Jan 26;9:5. doi: 10.1186/s13100-018-0109-4 (PMC5785829; doi:10.1186/s13100-018-0109-4)
Supplement: Supplementary file 4 — This file contains Tables S1 – S5. (PDF 52 kb) [file 13100_2018_109_MOESM4_ESM.pdf]

Table S1. Pairwise sequence comparison of T4SS proteins encoded by *tfs, com, cag* and *A. tumefaciens* pTi.

a.

	**Percentage sequence identity(similarity) from pairwise sequence comparison of T4SS proteins**											
**T4SS protein**	***A. tumefaciens* homologue[1] vs**			**Cag homologue[2] vs**			**Com homologue[2] vs**			**L1C1R1[3] vs L2C2R2**	**L1C1R1[3] vs Tfs3**	**L2C2R2[3] vs Tfs3**
	**L1C1R1 (G27)**	**L2C2R2 (R036d)**	**Tfs3 (Gambia[4])**	**L1C1R1 (G27)**	**L2C2R2 (R036d)**	**Tfs3 (Gambia[4])**	**L1C1R1 (G27)**	**L2C2R2 (R036d)**	**Tfs3 (Gambia[4])**			
**VirB2**	17.3(37)	19.3(31.9)	17.7(30.8)	18.5(34.6)	17.6(32.4)	18.8(29.7)	28.7(45.5)	20.9(33.6)	54.2(71.9)	40.6(59.4)	25.7(44.6)	25.2(40.8)
**VirB3**	<15	15.7(25.6)	<15	-	-	-	44.8(59.8)	38.3(56.4)	62.1(82.8)	51.1(72.7)	39.1(58.6)	38.3(57.4)
**VirB4**	21.3(38.2)	19(37.6)	19.5(37)	18.5(33.7)	17.4(32.4)	19.3(34.9)	34.9(54.2)	35.8(57.1)	45.5(63.1)	42.7(62.1)	35.3(54.5)	36.6(57.1)
**VirB6**	16.7(32.9)	<15	<15	15.1(25.2)	<15	<15	-	-	-	33(48.5)	21.1(35)	20.9(37.6)
**VirB7**	18.3(31.7)	21.7(31.7)	17.7(27.4)	<15	<15	<15	27.3(45.5)	42.5(57.5)	37.5(50.0)	51.2(60.5)	33.3(45.8)	36.2(44.7)
**VirB8**	15.6(30.4)	<15	<15	16.2(32.6)	<15	<15	30(46.3)	31.8(47)	28.3(43.6)	41.1(55.4)	27.5(43.5)	29.3(46.5)
**VirB9**	<15	<15	<15	<15	<15	17.7(30.6)	26.4(38.8)	27.8(39)	29.6(38.4)	96.7(98.1)	23.4(32.7)	23.6(34)
**VirB10[5]**	<15	17.8(29.8)	17.5(31.0)	19.7(32.2)	19.5(31.9)	21.3(34.5)	43.4(59.2)	38.9(52.6)	49.6(61.7)	75.6(82.9)	45.6(60.4)	41.3(57.6)
**VirB11**	27.1(40.9)	21(39.4)	26.1(43.1)	22.9(41.2)	27.1(47.9)	27.6(45.6)	-	-	-	54.1(70.6)	43.7(60.2)	40.7(62.4)
**VirC1**	22.8(39.6)	22.2(39.5)	20.7(33.3)	-	-	-	-	-	-	98.2(99.1)	64.9(79.3)	65.3(79.3)
**VirD2**	<15	<15	<15	-	-	-	-	-	-	33.1(49.4)	23.5(40.3)	22.9(36.7)
**VirD4**	17.1(25.4)	17.6(29.8)	17.5(32.1)	17.9(32.7)	19.6(36.1)	18.8(33.6)	-	-	-	51.2(64.4)	41.1(55.3)	37.8(54.6)

b.

	**Percentage sequence identity(similarity) from pairwise sequence comparison of T4SS proteins with the conserved *H. pylori* orphan VirB11**											
**T4SS**	***A. tumefaciens* C58 pTi VirB11**			**Cag VirB11**			**Com**			**Tfs4** (L1C1R1)	**Tfs4** (L2C2R2)	**Tfs3**
**Strain origin of orphan VirB11**	**G27**	**R036d**	**Gambia[4]**	**G27**	**R036d**	**Gambia[4]**	**G27**	**R036d**	**Gambia[4]**	**G27**	**R036d**	**Gambia[4]**
**Sequence identity**	21.4(37.6)	21.5(37.2)	21.4(36.3)	27.4(46.7)	26.7(46.2)	27.1(47.0)	-	-	-	39.1(57.9)	39.5(60.2)	52.1(71.6)

[1]Prototypical T4SS proteins encoded by the pTi plasmid of *A. tumefaciens* strain C58 were used for all comparisons.
[2]Pairwise sequence comparisons were between Cag, Com and Tfs homologous protein sequences from the same strain background (as indicated).
[3]Distinct Tfs4 modular types L1C1R1 and L2C2R2 as defined in this study.
[4]'Gambia' refers to reference strain Gambi94/24
[5]Pairwise sequence comparison of the Cag VirB10 homologue (CagY) only considered sequence within the C-terminal 357 amino acids comprising the VirB10 domain of the CagY protein.

Table S2. Distribution of *tfs4* ICE types within different *H. pylori* phylogeographic populations

		**Prevalence (%) of different *tfs4* ICEs in *H. pylori* populations**								
**Population**	**Strains (*tfs4*+)[1]**	**L1C1R1**	**L2C2R2**	**L2C1R1**	**L2C1R2**	**L2C1R1f**	**L1C1R1f**	**LmC1R1**	**LmC1R2**	**LmC2R2**
**hpEurope**	48	8 (17)	5 (10)	1 (2)	2 (2)	3[b] (6)	1 (2)	1 (2)	1 (2)	-
**hpAfrica1**	85	12[a] (14)	2 (2)	1 (1)	-	31[d] (37)	2 (2)	1 (1)	-	-
**hpAfrica2**	3	3[b] (100)	-	-	-	-	-	-	-	1[a] (33)
**hpAsia2**	6	2 (33)	-	-	-	-	-	-	-	-
**hspEAsia**	20	6 (30)	2 (10)	-	-	-	-	-	-	-
**hspAmerind**	8	6[b] (75)	-	-	-	-	-	-	-	-
**Totals**	170	37 (22)	9 (5)	2 (1)	2	34 (20)	3 (2)	2 (1)	1	1

[1]strains harbour either intact or remnant *tfs4* ICEs. P value was determined by Fisher's Exact Test and indicates significant association (positive or negative) of a *tfs4* cluster region with a particular *H. pylori* population. [a]P<0.05, [b]P<0.01, [c]P<0.001, [d]P<0.0001.

Table S3. Diversity of selected *tfs*, *com* and *cag* sequences

**Gene cluster**	***H. pylori* gene**	***vir* gene homologue**	**Minimum % sequence identity (nucleotide)**	**Minimum % sequence identity (amino acid)**	**Distinct variants**
tfs3 Left segment *tfs3* Central	t3_C9	*virD2*	85.33	85.5	1
	t3_C1	*xer*	90.1	92.96	1
	t3_V20	-	82.89	79.4	2
	t3_V21	-	78.48	79.49	2
	t3_V1	-	75.44	70.8	2
	t3_V24	-	43.44	18.36	4
	t3_C3	-	68.51	61.5	5
	t3_C2	*virB6*	62.72	52.71	4
	t3_C20	-	84.8	84.49	1
	t3_C6	-	84.44	81.98	1
	t3_V25	-	78.18	62.96	3
	t3_C7	*virC1*	87.0	87.11	1
tfs3 Right segment	t3_C11	*virB11*	91.53	92.36	1
	t3_C12	*virB10*	88.59	86.82	1
	t3_C13	*virB9*	86.5	84.63	1
	t3_C14	*virB8*	83.68	80.85	1
	t3_C17	*virB4*	90.65	94.06	1
	t3_V36	-	66.67	50.68	2
	t3_C18	*virB3*	87.5	87.36	1
	t3_C21	-	87.71	87.02	1
tfs4 L1/L2	t4_C1	*xer*	61.02	53.82	2
	t4_V1	-	79.68	71.37	2
	t4_C3	-	52.53	35.65	2
	t4_C2	*virB6*	43.16	23.01	2
	t4_C6	-	45.74	25.5	2
tfs4 C1/C2	t4_C7	*virC1*	91.78	95.41	1
	t4_C9	*virD2*	53.04	35.59	2
	t4_C11	*virB11*	60.62	54.84	2
	t4_V17	-	92.18	88.6	1
	t4_C12	*virB10*	79.19	66.5	2
	t4_C13	*virB9*	93.19	91.85	1
tfs4 R1/R2	t4_C14	*virB8*	61.25	51.41	2
	t4_C17	*virB4*	55.62	43.3	2
	t4_C18	*virB3*	56.7	47.67	2
	t4_C21	-	47.11	32.14	2
com	*comB3*	*virB3*	92.8	93.1	1
	comB4	*virB4*	90.65	94.03	1
	comB8	*virB8*	88.89	89.88	1
	comB9	*virB9*	85.45	84.01	1
	comB10	*virB10*	91.69	93.35	1
cag	*cag1/zeta*	-	94.51	90.43	1
	cag4/gamma	-	85.29	87.57	1
	cag5/beta	-	93.28	97.19	1
	cag7/Y	*virB10*	85.31	84.13	1
	cag8/X	*virB9*	96.0	97.12	1
	cag12/T	-	95.37	97.14	1
	cag17/N	-	94.74	90.2	1
	cag23/E	*virB4*	96.04	97.55	1
	cag26/A	*cagA*	83.04	77.12	2
Other	g_C11	*virB11*	91.15	94.41	1
	g_V1	-	88.3	83.26	1

Table S4. Phylogeographic distribution of *tfs3* allelic types

Population	Strains (*tfs3*+)[2]	Prevalence (%) of *tfs3* allelic type[1] in *H. pylori* populations				
		**1111**	**2222**	**4531**	**4533**	**3443**
**hpEurope**	34	17 (50)	-	-	-	3 (9)
**hpAfrica1**	53	35[c] (66)	-	4[a] (8)	1 (2)	1 (2)
**hpAfrica2**	0	-	-	-	-	-
**hpAsia2**	5	-	1 (20)	-	-	-
**hspEAsia**	14	-	5[d] (36)	-	-	-
**hspAmerind**	5	-	-	-	2[b] (40)	2[a] (40)
**Totals**	111	52 (47)	6 (5)	4 (4)	3 (3)	6 (5)

[1]Each digit in the four digit code corresponds to a distinct clade determined from phylogenetic Neighbour-joining analysis of individual genes, t3_V24, t3_C3, t3_C2 and t3_V25.

[2]Strains harbour either intact or remnant *tfs3* ICEs

P value was determined by Fisher's Exact Test and indicates significant association (positive or negative) of a *tfs3* allelic type with a particular *H. pylori* population. [a]$P<0.05$, [b]$P<0.01$, [c]$P<0.001$, [d]$P<0.0001$ compared with indicated MLSTs.

Table S5. Pairwise comparison of concatenated MLST sequences and defined *tfs* ICE segments from different *Helicobacter* species.

	**Average % sequence identity (range) from pairwise sequence comparison of different *Helicobacter* species**				
**Comparators**	**MLST sequence[1]**	**t3_C9-C1[2]**	**t3_C9-C16[3]**	**t3_V33-V35[4]**	**t4_V4f-C9f[5]**
H. pylori/*H. pylori*[6]	95.9	89.1 (83-99.7)	86.8 (80-99.3)	92.2 (88-99.7)	93.9 (91-99)
H. pylori/hpAfrica2	91.8	n/a	n/a	n/a	93.13 (91-94.3)
hpAfrica2/*H. acinonychis*[7]	89.9	n/a	n/a	n/a	93.2[7]
H. pylori/*H. acinonychis*	87.7	n/a	n/a	n/a	93.8 (91-96.6)
H. pylori/*H. cetorum*[8]	81.3	85.3 (83-86.7)	82.2 (79-85.3)	86.2 (84-88)	n/a
H. pylori/*H. suis*	68.0	88.1 (83-91.7)	87.8 (81-94.3)	91.9 (87-98.2)	n/a
H. cetorum[8]/*H. suis*	67.9	86.9	84.3	84.2	n/a

[1]pairwise comparison of concatenated MLST sequences, representing 179 *H. pylori* strains from all populations (including 3 hpAfrica2 strains), *H. cetorum* strains MIT 00-7128 and MIT 99-5656, *H. suis* strain HS1 and *H. acinonychis* str. Sheeba.
[2]pairwise comparison of *tfs3* ICE sequences from strains UM114, P-13, P12, Gambia94/24, A-5, P-41, India7, CPY6311, OK310, H-29, Shi112, Akalvik117, Puno135, Shi417, H-3, SJM180, *H. cetorum* MIT 00-7128 and *H. suis* HS1.
[3]pairwise comparison of *tfs3* ICE sequences from all strains noted in footnote[2] except P12, and additionally including PeCan18b.
[4]pairwise comparison of *tfs3* ICE sequences from strains Shi112, Shi417, Aklavik117, India7, CPY6311, OK113, UM114, B8, P12, Gambia94/24, P-41, Pecan18b, *H. cetorum* MIT 00-7128 and *H. suis* HS1.
[5]pairwise comparison of *tfs4* ICE sequences from strains 51, Shi470, Cuz20, SouthAfrica7, 26695, PeCan4, SNT49, G27, P12, A-11, GAM249T, GAM265BSii, Gambia94/24, A-8, NQ4053, P-25 and *H. acinonychis* str. Sheeba. Sequences equivalent to the entire *tfs4* remnant present in *H. acinonychis* including fragments (f) of the flanking genes indicated
[6]pairwise comparison of sequences representative of *all H. pylori* MLST populations except hpAfrica2.
[7]pairwise sequence comparison between *H. pylori* strain SouthAfrica7 and *H. acinonychis* str. Sheeba.
[8]Strain *H. cetorum* MIT 00-7128.
n/a – not applicable
